# Assessing the process performance of ballistic separators in packaging waste sorting plants using sensor-based process monitoring

**DOI:** 10.1177/0734242X261433694

**Published:** 2026-04-11

**Authors:** Alena Maria Spies, Tabea Scherling, Annika Ludes, Bastian Küppers, Nils Kroell, Karoline Raulf, Kathrin Greiff

**Affiliations:** 1 Chair of Anthropogenic Material Cycles (ANTS), RWTH Aachen University, Germany; 2 STADLER Anlagenbau GmbH, Altshausen, Germany

**Keywords:** mechanical processing, process optimisation, process automation, material recovery facility, lightweight packaging waste, paper and board recycling, circular economy

## Abstract

To effectively utilise secondary raw materials, pre-concentrates of defined quality are required. Packaging waste streams are treated in waste sorting plants, where different sorting operations are applied. Ballistic separators are frequently used to separate 2D and 3D materials during (pre)conditioning and therefore have a major influence on the overall process performance. Despite their widespread use, until now, few studies have examined how parameter settings affect the performance of ballistic separators. To assess and optimise process performance, we conducted a parameter study on packaging waste using an industrial-scale ballistic separator and near-infrared-based process monitoring. At 22.5 wt% 2D input content, we varied 2D material properties, paddle angle (15°, 17.5°, 20°), rotational speeds (*s*_1_–*s*_5_) and paddle types (45 mm, closed). All parameters showed significant effects on the process performance. In the material stream used, which contained a proportion of fine material, the 45 mm paddles demonstrated higher separation performance compared to the closed paddles. A specific rotational speed must be reached to achieve an effective separation efficiency. Beyond this, a further increase does not lead to measurable improvements. Two optimisation strategies were developed to determine process optima and enable the integration of the generated results into operational practice. Therefore, the results contribute to the optimisation and potential automation of ballistic separators and the overall improvement of packaging waste sorting plants.

## Graphical abstract

**Graphical abstract:**
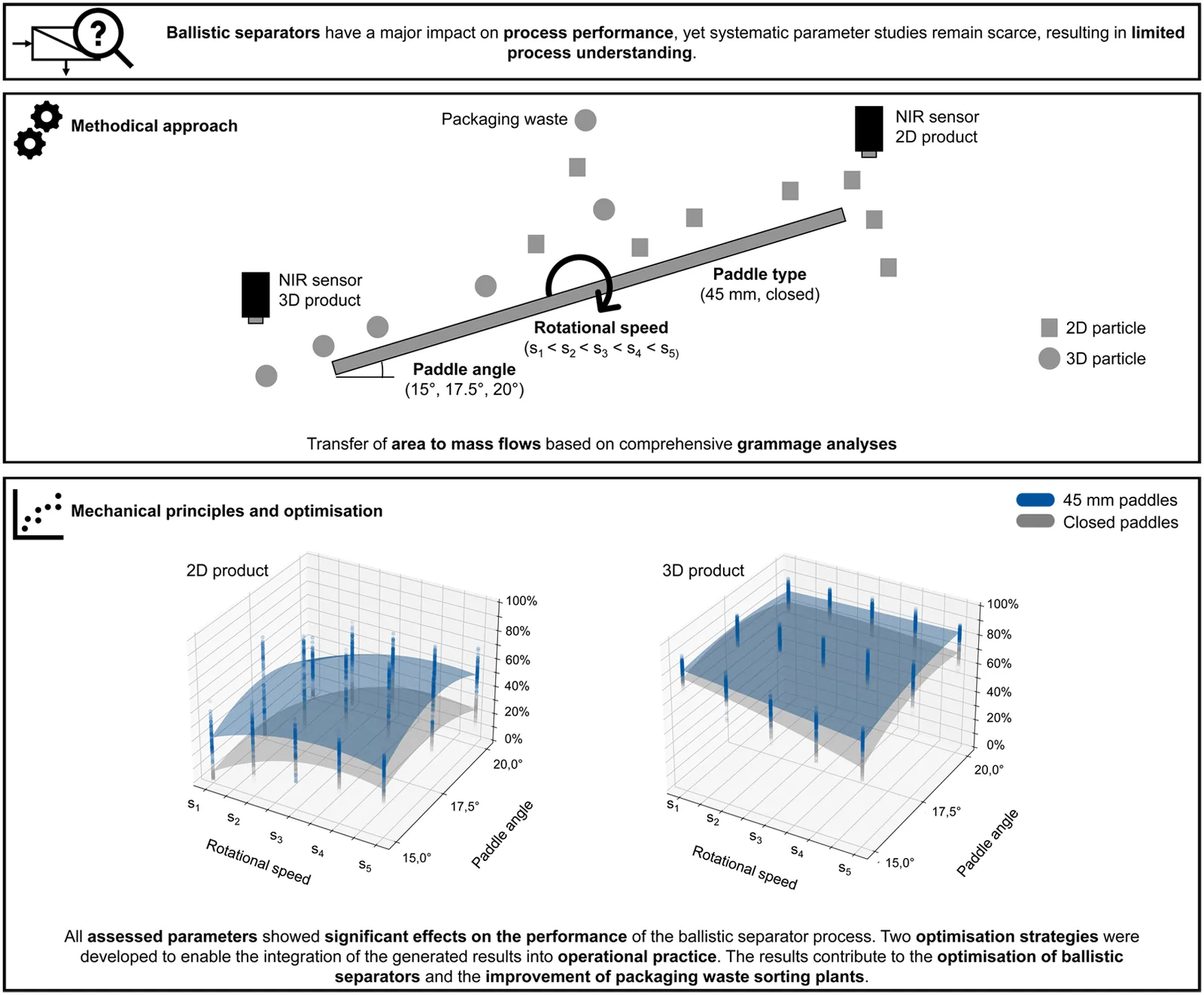

## Introduction

In the context of a circular economy, waste can be used as a secondary raw material to protect natural resources (Bundesministerium für Umwelt, Naturschutz, nukleare Sicherheit und Verbraucherschutz, 2024). To effectively integrate secondary raw materials into recycling chains, pre-concentrates must meet specific quality definitions. Therefore, pre-concentrates are generated from the collected waste stream by waste sorting plants (Pretz et al., 2020). Waste sorting plants consist of individual process step combinations, including, for example comminution, (pre)conditioning and sorting (Kranert, 2024). The produced pre-concentrates can be forwarded to other plants for material recycling or energetic recovery (Pretz et al., 2020). In this context, automated upstream and downstream sorting processes could enable more sustainable waste management (Salem et al., 2023).

### Ballistic separator

#### Operation principle

A relevant step in waste sorting and processing plants is the separation of 2D and 3D materials, commonly achieved using ballistic separators. Ballistic separators consist of paddles with individually rotating longitudinal elements (so-called paddle segments; Feil and Pretz, 2020). Due to their material characteristics, rollable 3D particles are ejected at the lower end of the paddles (Feil and Pretz, 2020). Flat 2D particles are ejected at the upper end (Feil and Pretz, 2020). Multiple paddles enable a higher discharge speed of the 2D material compared to just one paddle in the separation layer of the ballistic separator (Sigmund, 2018). The separation is based on shape, elasticity, size and weight (resp., density) of the particles (Martens and Goldmann, 2016). Adjustable parameters of the ballistic separator are the input position of the material, the grid size (paddle type), the rotational (oscillating) speed and the paddle angle (Eule, 2013).

#### Applications in waste sorting plants

Depending on the process configuration and the waste stream, ballistic separators are integrated at various positions in the process chain of waste sorting plants. In material recycling facilities, they are often positioned after sieving by a drum screen to produce pre-conditioned material for further sorting steps (Sigmund, 2018). In packaging waste sorting plants, ballistic separators are typically used to separate films and papers from 3D plastics during (pre)conditioning (Feil et al., 2021) or during the sensor-based sorting (SBS) cascade (Knappe et al., 2021). For SBS, the separation of 2D materials is particularly important (Feil et al., 2021). Küppers et al. (2021) have demonstrated, for example, that 5 wt% 2D material content in the SBS input at high throughput rates has a similar effect on the target material yield as a malfunction of 20% of the air valves. Studies examining the modelling of entire packaging waste sorting plants have shown a significant influence of the ballistic separator on overall process performance (Kleinhans et al., 2021; Tanguay-Rioux et al., 2022). Kleinhans et al. (2021) demonstrated, within a use case of their model, that the performance of a ballistic separator could be increased through optimised parameter settings after a certain period of operation. Tanguay-Rioux et al. (2022) showed that process performance could be improved by enhancing the separation efficiency of the second ballistic separator in the investigated plant, for example, by adjusting the operating conditions.

#### State of the art to assess the process performance

Although the first concept of ballistic separation was patented in 1978 (Forslund, 1978), and ballistic separators are widely applied in waste sorting and processing plants (cf. section ‘Applications in waste sorting plants’), there have been few studies on the influence of parameter settings on process performance. Sigmund (2018) stated that a higher paddle angle has a negative impact on the yield of the 2D material in the 2D product and a positive impact on the yield of the 3D material in the 3D product. The purity exhibits the opposite behaviour compared to the yields (Sigmund, 2018). Several studies focused on the potential use of ballistic separators for processing landfill mining material. To treat landfill mining material, including construction and demolition waste, STADLER Anlagenbau GmbH (Altshausen, Germany) developed an adapted ballistic separator (type STT6000; Mielke, 2018). García López et al. (2018) tested the STT6000 with landfill mining material to produce refused-derived-fuel (RDF). The generated 2D material flows showed a promising material composition for use as RDF (García López et al., 2018). In further work, two ballistic separators were combined at the start of a processing chain (García López et al., 2019). The results show that construction and demolition waste was mainly found in the 3D products, whereas other municipal solid waste materials were accumulated in the 2D products (García López et al., 2019). As a large proportion of fines (<90 mm) accumulated after the second ballistic separation step, further processing of this fraction was investigated (Hernández Parrodi et al., 2019).

For the processing of mixed commercial and mixed municipal solid waste, Möllnitz et al. (2021) investigated the influence of a drum screen in front of a ballistic separator STT5000_6_1. The paddle angle was set to 12.5° to produce a 3D product with high purity (Möllnitz et al., 2021). The results showed an increased overall screening efficiency when a drum screen was used before the sieving with the ballistic separator (Möllnitz et al., 2021). Moreover, the results indicate that pre-screening leads to an overall improved process performance of the ballistic separator (Möllnitz et al., 2021). Lasch et al. (2025a) investigated the mobile Ballistor 4300 (Komptech GmbH, Frohnleiten, Austria) using mixed commercial waste, testing the parameters paddle speed, paddle angle and air supply. However, they already noted that mixed commercial waste is not directly comparable to the material used in this study, citing both the preprint of this work (Spies et al., 2024c) and conference proceedings that presented a small subset of results from these trials (Spies et al., 2024b). However, for packaging waste, the impact of varying ballistic separator parameters on process performance has, to the best of the authors’ knowledge, not yet been comprehensively investigated.

### Process monitoring and optimisation

Several studies have already demonstrated that sensor-based material flow monitoring (SBMM) can yield reliable results in packaging waste sorting plants. Schlögl et al. (2022) reported initial results on the application of SBMM in a packaging waste sorting plant. Kroell et al. (2024a) developed an near-infrared (NIR) based quality monitoring approach for plastic pre-concentrates in lightweight packaging waste (LWP) sorting plants. In addition, Schlögl et al. (2024) demonstrated the potential to use data from sensor-based sorters and compared these with data obtained from external sensors. However, although the topic of SBMM has already been investigated, few studies in waste sorting and processing applications have focused on sensor-based process monitoring (SBPM), even though continuous process monitoring allows several repetitions per parameter setting and does not require sampling or manual analysis (Kroell et al., 2022). Examples of parameter studies using SBPM include, in addition to the previously mentioned study on a ballistic separator for the processing of commercial waste (Lasch et al., 2025a), the evaluation and optimisation of shredding mixed commercial waste (Khodier et al., 2021), where the data were subsequently used for time series analyses (Lasch et al., 2025b). However, the predictable time windows were slightly too short to implement dynamic process control (Lasch et al., 2025b). Further parameter studies using SBPM have addressed the sieving of LWP by a drum screen (Chen et al., 2023) and the sorting of polyethylene terephthalate (PET) from a 3D plastic fraction by a sensor-based sorter (Kroell et al., 2024b). Kroell et al. (2024b) also developed a process model that provides a time-resolved prediction for the investigated sorting operations. SBPM can thus support improved process understanding and optimisation, whereas continuous SBPM can ultimately contribute to manual or automated process control (Küppers et al., 2022).

In addition to individual process evaluation, integration into process chains must be considered. Different approaches have been employed to model plastic waste sorting plants. Tanguay-Rioux et al. (2022) developed a model of a Canadian material recovery facility. Kleinhans et al. (2021) developed a predictive modelling approach for a household packaging waste sorting plant. Parbat et al. (2024) used the mass balance model developed by Kleinhans et al. (2021) to design an architecture for a digital twin of a sorting plant. Emmert et al. (2024) proposed the use of Artificial Neural Twins to model process chains for bulk material processes in plastic recycling. For the modelling of mass flows (Brunner and Rechberger, 2016), as well as of processing plants, transfer coefficients (TCs, also referred to as split factors or separation parameters) are often required (Kleinhans et al., 2021; Tanguay-Rioux et al., 2022). Although TCs have a significant influence on the recovery potential of processing plants (Tanguay-Rioux et al., 2021), their determination has so far mainly been based on performance specifications provided by sorting equipment manufacturers (which usually underestimate performance to ensure compliance with given performance guarantees), on modelling the physical forces acting on particles (which represent real waste only to a limited extent) and on mass balances based on sampling (which involve a certain sampling error) (Kleinhans et al., 2021). In this context, SBPM provides a new method for determining TCs.

In conclusion, SBPM enables continuous process monitoring and comprehensive parameter studies. The resulting process understanding may support optimisation at both the process and process chain levels, while continuous monitoring can provide a basis for process control and automation.

### Study objective and research questions

Ballistic separators are commonly used in packaging waste sorting plants and have a significant impact on the overall process performance (cf. section ‘Applications in waste sorting plants’). To date, the influence of ballistic separator parameter settings on process performance has not been comprehensively studied in the context of packaging waste (cf. section ‘State of the art to assess the process performance’). To address this research gap and ultimately support process optimisation through improved process understanding, we conducted a parameter study on an industrial-scale ballistic separator (STT2000; STADLER Anlagenbau GmbH). As SBPM enables comprehensive parameter studies and continuous process monitoring, thereby supporting process optimisation and potentially enabling a development towards process control and automation (cf. section ‘Process monitoring and optimisation’), we used NIR-SBPM to monitor the product streams. However, with this method, only area flows were detected. To obtain mass-based process indicators, comprehensive grammage analyses were conducted. To quantify the influence of material characteristics on process performance, the 2D material properties resulting from different paper and board (PB) fractions and the 2D material contents were varied. As paddle type, paddle angle and rotational speed are key parameter settings influencing the process performance of ballistic separators, these parameters were systematically varied. For all parameter settings, the impact on purities and yields of 2D and 3D materials in the 2D and 3D products was determined. Based on the generated data, optimised operating points were identified. The objective of this study is to improve the understanding of ballistic separator operation in packaging waste sorting plants to support process optimisation and automation. From the study objective, we derive the following research questions (RQs):

**RQ 1:** How can the area flows be converted into mass flows, and how does the conversion from area to mass flows influence the results?
**RQ 2:** How do the 2D material properties (RQ 2.1), the paddle angle (RQ 2.2), the paddle type (RQ 2.3), the rotational speed (RQ 2.4) and the 2D material content (RQ 2.5) influence the separation of 2D- and 3D-material fractions?
**RQ 3:** How can a process optimum be determined, and what are optimal process parameters?

## Materials and methods

The trials were conducted in March 2023 at the Slovenian Test and Innovation Centre of Willi Stadler d.o.o. The test material was prepared, and the process was monitored using different NIR-sensors.

### Test material

#### Test material composition

**Figure 1.**
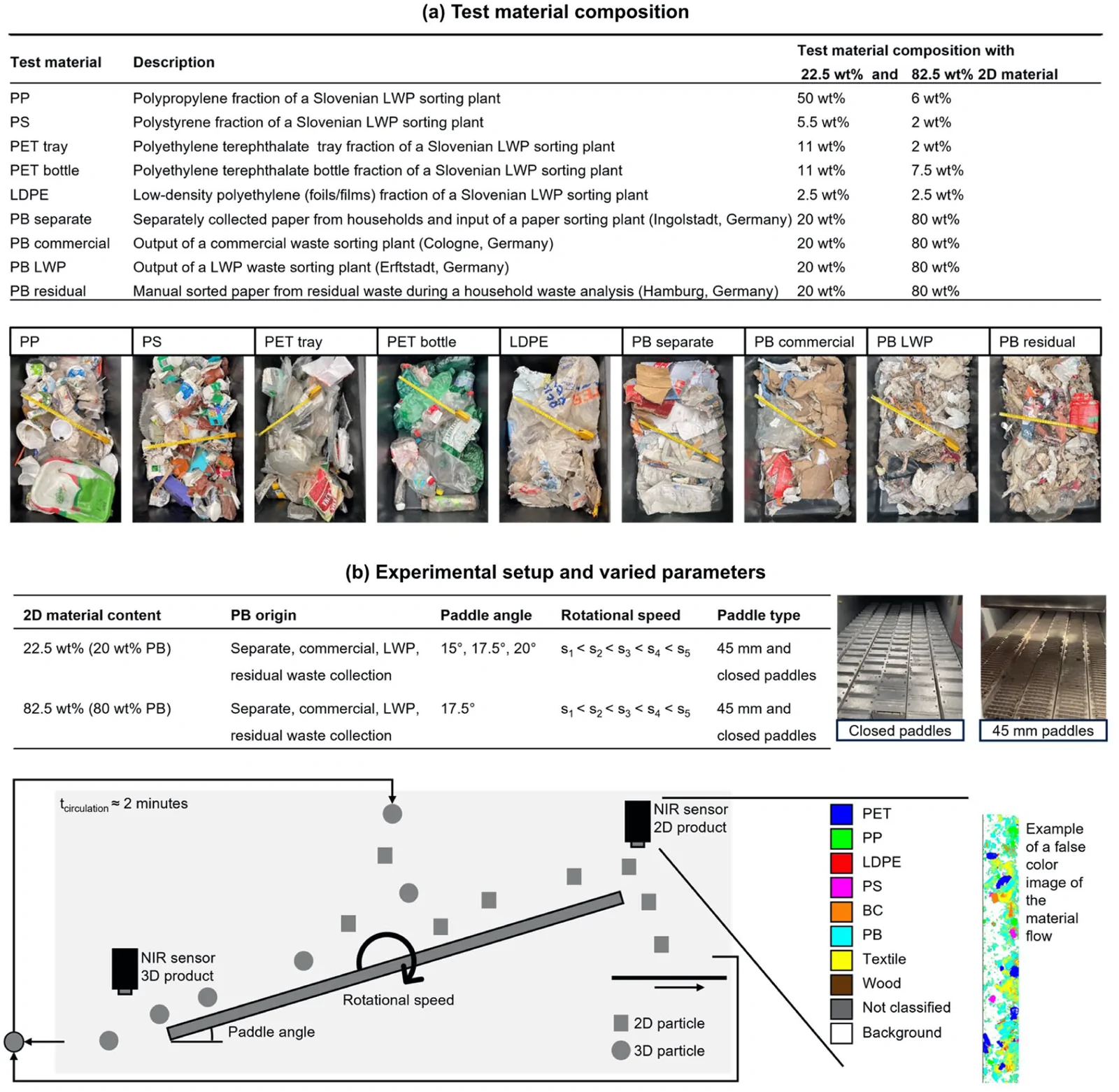
Methodical approach for the conducted parameter study on optimisation of a ballistic separator (a) test material compositions (for each experiment, one PB type was used) (b) experimental procedure, varied parameters and NIR recorded material fractions with exemplary false colour image. NIR: near-infrared; PB: paper and board.

To enable a clear classification of material and particle characteristics, test material charges with defined material compositions were prepared. The test material was sampled at waste accumulation sites. For each trial, 100 kg of test material was prepared using real waste material. Polypropylene (PP), PET bottles, PET trays and polystyrene (PS) were used as 3D materials, whereas low-density polyethylene (LDPE, foils/films) and PB were used as 2D materials. Beverage cartons (BCs) should usually be ejected into the 3D product in LWP sorting plants (Feil et al., 2016). However, BCs contained in the test material had mainly 2D material properties and were therefore classified as a 2D material. Wood was classified as a 3D material. The exact test material compositions and origins are provided in Figure 1(a). Bulk densities for typical LWP product fractions have been determined in previous work (Spies et al., 2024a). The following densities can be assumed: PP 33 kg m^−3^, PET bottles 44 kg m^−3^, PET trays 29 kg m^−3^, PS 32 kg m^−3^ and LDPE 12 kg m^−3^ (Spies et al., 2024a).

To vary the 2D material content, two mixtures with different PB contents were prepared. The first test material consisted of 20 wt% PB and reflected the composition before SBS cascades in LWP sorting plants. The second test material consisted of 80 wt% PB. It reflected the composition of PB streams from which plastics must be separated (e.g. in paper sorting plants or as a cleaning step of the PB product stream in LWP sorting plants).

Even though NIR-based differentiation between high-density polyethylene (HDPE) and LDPE is possible on a laboratory scale (Sato et al., 2003), differentiation within a material mixture is challenging. Therefore, PE (polyethylene) was used only as LDPE to enable precise classification of material and particle characteristics. To vary 2D material properties, PB from different origins (separate paper collection, commercial, LWP and residual waste) was used. Each PB material was manually analysed. The sorting catalogue used, the compositions and the particle size distributions of the PB fractions are given in Supplemental Material 1.

#### Test material grammages

Since SBPM was used to monitor the material flows, the generated results represent area flows. However, in the waste management sector, process evaluations are generally based on mass measurements. To convert the measured area flows into mass flows, grammages ρ_*A*_,_*i*_ are usually determined and calculated according to equation (1) with the total mass *m_i_* and the total area *A_i_* of each recorded material fraction (Kroell et al., 2024a).

(1) $$\rho_{A,i}=\frac{m_{i}}{A_{i}}$$

Although there have been investigations (Kroell et al., 2023, 2024a), the conversion from area to mass flows depends strongly on the used material. In this study, the material consisted of real waste articles with material combinations (e.g. labels, sleeves, lids, covers). To determine the grammages, the test material was divided into 157 subsamples. Each subsample was weighed and recorded using an NIR sensor. This enables the determination of grammage variances. The grammage determination was conducted using the entire test material, which was the same material used during the trials. Two options were identified to calculate the resulting grammages. The following notation is used:

- *i*: Physical test material divided into subsamples.
- *j*: Material class detected by NIR-sensor, corresponding to the test material *i*.
- *N*: Number of subsamples whereby *N*(*i*) represents the total number of subsamples for the test material *i* and *N* represents the total number of subsamples for all materials.
- Option 1: For each test material *i*, the mass per subsample *m_n_*,_*i*_ is divided by the recorded area per subsample, *A_n_*,_*i*_. The grammage ρ_*A,i*,1_ is determined by calculating the mean value of the grammages per subsample across all subsamples per test material *N*(*i*) according to equation (2).

(2) $$\rho_{A,i,1}=\frac{1}{N(i)}\sum_{n=1}^{N(i)}\frac{m_{n,i}}{A_{n,i}}$$

Option 2: For each test material *i*, the area $A_{n,i,j}$ correctly detected by NIR is divided by the area per subsample $A_{n,i}$ and then multiplied with the subsample mass $m_{n,i}$ . This ‘weighed’ mass per material is summed over all subsamples *N* and divided by the total area of the test material across all subsamples according to equation (3). This approach weighs all materials as they are detected and considers the presence of labels, sleeves or lids. Therefore, option 2 is particularly suitable if the material for which the grammages were determined is also used for conducted trials.

(3) $$\rho_{A,i,2}=\sum_{n=1}^{N}(\frac{A_{n,i,j}}{A_{n,i}}*m_{n,i}):\sum_{n=1}^{N}A_{n,i,j}$$

### Experimental setup

For the trials, a ballistic separator type STT2000 from STADLER Anlagenbau GmbH (2026) with six paddles was used. A detailed construction drawing of the ballistic separator, including dimensions, is provided in Supplemental Material 2. The investigated ballistic separator is commonly used in this configuration in LWP sorting plants. The experimental setup, including the material input and output streams, is illustrated in Supplemental Material 3. The same ballistic separator with the described setup was used for all experiments.

#### Investigated parameter settings

Throughout the conducted experiments, the constructive, mechanical characteristics of the ballistic separator (e.g. the number of paddles and the material feed position) were not altered. The ballistic separator parameters that were varied were the paddle angle, the rotational speed and the paddle type. The selected parameters, paddle angle, rotational speed and paddle type, represent adjustable operating parameters that can be modified in existing ballistic separators. In particular, the paddle angle and the rotational speed theoretically allow for adaptive control even during ongoing process operation. For the paddle angle and rotational speed, parameter ranges were selected that had shown relevant performance changes in preliminary trials with the investigated test material. These ranges, therefore, represent the maximum operational window within which a ballistic separator of type STT2000-6 can be operated with the given material composition under the applied boundary conditions (e.g. fines content, moisture, throughput and machine setup). Three different paddle angles (15°, 17.5° and 20°) and five rotational speeds of the paddles (*s*_1_ < *s*_2_ < *s*_3_ < *s*_4_ < *s*_5_) were varied. As paddle types, 45 mm paddles (paddles with 45 mm openings) and closed paddles were chosen. Pictures of the used paddle types are given in Supplemental Material 4. Both paddle types are already used in existing LWP sorting plants. Consequently, all operational parameters investigated are, in principle, transferable to other ballistic separators type STT2000 with six paddles. However, material-specific properties must also be considered.

All parameters were varied for the test material containing 22.5 wt% 2D material. For the test material containing 82.5 wt% 2D material, the paddle angle was fixed at 17.5°. For the 2D material fraction, only the paper content was varied, whereas the film content remained unchanged (cf. also Figure 1(a)). The material was processed in a loop system and used different times. Therefore, the material can be considered air-dry. However, several parameters that may affect process performance were not examined. Throughput was kept constant, and thus fluctuations expected under real-world conditions were not analysed. Moreover, the influence of fines and varying moisture contents were not examined systematically (cf. also section ‘Data adjustments and study limitations’).

Based on the varied ballistic separator parameters and the selected material mixtures, a total of 160 parameter setting combinations and, therefore, 160 independent trials were conducted. An overview of the varied parameters is given in Figure 1(b).

#### Experimental procedure

The generated samples (cf. section ‘Test material’) were continuously fed to the ballistic separator within a closed-loop system. The experimental procedure for the loop system is shown in Figure 1(b). A photograph of the experimental setup, including the material input and output streams, is additionally provided in Supplemental Material 3. To ensure an equalised material flow, the test material was conveyed and distributed for several minutes. After changing a parameter, the recording was paused for a minimum of 1 minute to ensure a realistic adjustment of the material behaviour. As the experiments were conducted at a scale comparable to industrial applications, ambient conditions slightly varied during the trials, including changes in relative humidity and temperature. With a circulation time of 2 minutes and a test material mass of 100 kg, a throughput of approximately 3 t hour^−1^ was aimed. The evaluated recorded sensor data suggest a slightly lower throughput of 2.54 t hour^−1^ (standard deviation over all trials of 0.25 t hour^−1^). Each parameter setting was kept for 6 minutes. With 160 trials and a trial duration of 6 minutes, 16 hours of sensor data were recorded and analysed.

### Sensor-based process monitoring

#### NIR-sensors

To evaluate the trials, SBPM of the 2D and 3D products was conducted (cf. Figure 1(b)). NIR-sensors were used, specifically EVK HELIOS EQ32 hyperspectral cameras from EVK Kerschhaggl GmbH (Raaba, Austria), with a spectral resolution of 3.1 nm per channel, a wavelength range of 1016–1684 and 1016–1688 nm and a spatial resolution of ≈22.91 and ≈16.45 mm^2^ px^−1^ to record the 2D and 3D product, respectively. The recording was performed at a framerate of 450 Hz for the 2D NIR-sensor and 451 Hz for the 3D NIR-sensor. Pictures of the installed NIR-sensors are given in Supplemental Material 5 (2D) and Supplemental Material 6 (3D). During the trials, the throughput was kept constant. Operating conditions were such that no significant overlaps occurred in either the 2D or 3D outputs. All measurements were continuously monitored throughout the experiments. If any irregularity was detected (e.g. material accumulation beneath one of the sensors), the experiment was terminated, the issue was resolved and the experiment was restarted.

#### NIR classification

For NIR classification, the software EVK SQALAR (EVK DI Kerschhaggl Ltd., 2026) with the classification algorithm CLASS32 was used. The preprocessing and spectral processing included intensity calibration, spatial correction, bad pixel interpolation, normalisation and formation of the first derivation of the spectra (Küppers et al., 2019). Three wavelength ranges were chosen for classification in which the spectra differed significantly for the NIR-sensor of the 2D product (1120–1270, 1314–1468 and 1626–1684 nm) and 3D product (1120–1270, 1352–1474 and 1620–1688 nm). The background differentiation was achieved using the combined features mode, in which an EC3 algorithm and a threshold to the dynamic NIR spectra were applied. Representative particles of the test material (cf. Figure 1) were trained. The classifier was created and cross-checked by two people with many years of experience in creating NIR classifiers. The same particles were used for creating the classifier and tested using various particles from the test material. Therefore, it was assumed that the classifiers of both NIR sensors are comparable. As different PB fractions with impurity contents were used, materials with NIR spectra similar to the PB spectrum (textile, wood and BCs) were trained. Material used for training the NIR classifier is shown in Supplemental Material 7. To classify the material of which each specific article consists, labels and sleeves from articles were additionally trained. The resulting NIR spectra are given in Supplemental Material 8. Areas that could not be assigned to a trained spectrum were categorised as not classified (cf. Figure 1(b)). A programme developed at the Chair of Anthropogenic Material Cycles (ANTS), RWTH Aachen University, recorded the false-colour images (cf. Figure 1(b)).

#### NIR postprocessing

Area flows were derived based on the recorded false-colour images. Due to the technical process setup, the material ejected into the 3D product was detected in free fall (cf. Supplemental Material 6). To create comparability between the 3D NIR-sensor and the 2D NIR-sensor, an ideal round object detectable within the NIR range was passed through both sensors. The images were spatially calibrated so that the objects under both sensors had the same size and were ideally round. Not classified areas were neglected.

For data processing, Python scripts were developed using different open-source packages. The packages NumPy (Harris et al., 2020) and pandas (McKinney, 2010; The Pandas Development Team, 2022) were used for data processing, Matplotlib (Hunter, 2007) and seaborn (Waskom, 2021) for data visualisation, SciPy (Virtanen et al., 2020) for statistical evaluation and scikit-learn (Pedregosa et al., 2011) for curve fitting models.

### Data adjustments and study limitations

Since the results are based on empirically generated data, several factors can lead to limitations and should be considered when interpreting the data. The following outlines the limitations, along with the respective data validations and adjustments.

Limitations due to the loop system include a slightly longer circulation time for the 2D material (approximately 6 seconds). It is assumed that this difference is negligible. During the trials, material losses occurred when the material became tangled in the loop system (particularly PB) or fell off the belt. The material was constantly returned to the loop system. Nevertheless, an influence on the recorded area flows due to material losses or entangled material cannot be entirely excluded, at least temporarily. The effects are potentially higher for trials with 82.5 wt% 2D material content. Since the same test material was used for all tests to ensure the highest possible comparability, the water content and possibly also the material properties could have changed slightly. Since the change of parameter settings, especially for the paddle type and angle, required a high effort, it was not possible to arrange the tests randomly as suggested, for example, by Siebertz et al. (2010). To account for this, each parameter variation was evaluated in the context of all tested parameters (e.g. the data evaluated for angle settings included the different tested rotational speeds, material compositions and paddle types). As a result, the identified process principles can be considered reasonably robust, as they were observed across different times and parameter settings. However, because the focus of this publication is the identification of robust main effects, factor interactions were not investigated in either the statistical analysis or the graphical evaluation.

Another general limitation is that the material streams were measured using two different sensors. The NIR-sensors were spatially calibrated using a round object (cf. section ‘NIR postprocessing’). To validate this calibration, test material objects from each material fraction were passed through the 3D NIR-sensor (free fall) and another NIR-sensor above a belt (cf. Supplemental Material 9). The main material fractions (PET, PP, PS, PB) show approximately the same ratio when comparing the recorded areas from both sensors. However, for LDPE, almost twice as much material was detected from the 3D NIR in free fall due to the higher air resistance and longer fall time of LDPE. Since this effect is weakened within a material flow and the LDPE proportion of 2.5 wt% is relatively low, no further calibration was conducted. All unclassified area flows were excluded from the analysis results as they primarily resulted from belt pollution. An over-detection of textiles was noted. As it was not possible to determine the extent to which textiles were over-detected, the entire textile area flow was attributed to the PB area flow. This assumption was possible since the contained textiles originated from small PB impurity contents (mostly PB from commercial and LWP waste) and did not change the evaluation, as both materials were classified as 2D materials. In addition, during the development of the classification model, spectra of PB labels on PET trays were trained to achieve better PET material classification (cf. section ‘NIR classification’). Since the PB water content obviously changed during the trials, a portion of the PB was recognised as PB labels on PET and was thus classified as PET, especially in the 2D product. As it was not possible to determine the incorrectly detected PB proportion and the increase in the detected PET area flow was rather low, no further calibration was conducted. During the grammage recording, an over-detection of PP was noted. The over-detection resulted from a polluted background. To eliminate the over-detection, PP pixel clusters below a certain size were excluded from the grammage recordings. For PB grammages, no distinction was made between PB from different origins. As it was not possible to distinguish between PET trays and bottles based on the false colour images, an average grammage for PET was calculated.

In addition, some parameters that probably influence the ballistic separator process performance were not part of the investigation, and their influence should be quantified during further investigations. This includes the input point of the material on the paddle, the water content of the material, the throughput and differing material compositions (other 2D material contents and other materials than the used packaging waste). Additionally, the influence of fine material on process performance should be examined more thoroughly. In general, to demonstrate reproducibility and validate the results, experiments should be conducted in a real packaging waste sorting plant.

### Assessment of process performance

The trials were evaluated by purity *P* and yield *Y*. With the grammages per material type, the area flows were converted into mass flows (cf. section ‘Test material grammages’) and mass-based purities *P_m_* (equations (4) and (5)) and yields *Y*_m_ (equations (6) and (7)) were calculated. Therefore, the mass flows of the 2D/3D materials in the 2D/3D product ${\dot{m}}_{2D/3D,2D/3D-\mathrm{product}}$ the mass flows of the 2D/3D product and the total masses of all 2D/3D materials ${\dot{m}}_{2D/3D-\mathrm{total}}$ were used. As the purities and yields always refer to 2D material in the 2D product or 3D material in the 3D product, in the following, the terms 2D purity (4)/yield (6) and 3D purity (5)/yield (7) are used.

2D purity:

(4) $$P_{m,2D,2D-product}=\frac{{\dot{m}}_{2D,2D-product}}{{\dot{m}}_{2D-product}}$$

3D purity:

(5) $$P_{m,3D,3D-\mathrm{product}}=\frac{{\dot{m}}_{3D,\;3D-\mathrm{product}}}{{\dot{m}}_{3D-\mathrm{product}}}$$

2D yield:

(6) $$\begin{array}{l} Y_{m,2D,2D-product}=\frac{{\dot{m}}_{2D,2D-product}}{{\dot{m}}_{2D-total}}\\ =\frac{{\dot{m}}_{2D,2D-product}}{{\dot{m}}_{2D,2D-product}+{\dot{m}}_{2D,3D-product}} \end{array}$$

3D yield:

(7) $$\begin{array}{l} Y_{m,3D,3D-\mathrm{product}}=\frac{{\dot{m}}_{3D,3D-\mathrm{product}}}{{\dot{m}}_{3D-\mathrm{total}}}\\ =\frac{{\dot{m}}_{3D,3D-product}}{{\dot{m}}_{3D,2D-product}+{\dot{m}}_{3D,3D-product}} \end{array}$$

To characterise the process for a specific experiment *i*, transfer coefficients TC_*i*_ can be used (Brunner and Rechberger, 2016; Kleinhans et al., 2021). Since two product streams are involved (2D and 3D products), two TCs are calculated to fully characterise the process according to equations (8) and (9).

(8) $$TC_{2D,i}=\frac{{\dot{m}}_{2D,2D-product,i}}{{\dot{m}}_{2D,2D-product,i}+{\dot{m}}_{2D,3D-product,i}}$$

(9) $${\mathrm{TC}}_{3D,i}=\frac{{\dot{m}}_{3D,3D-product,i}}{{\dot{m}}_{3D,2D-product,i}+{\dot{m}}_{3D,3D-product,i}}$$

To visualise an optimum for the mass-based purity *P_m_* and yield *Y_m_* for each output stream (2D/3D product) with one process indicator, the separation efficiency η according to equation (10) is used (Bunge, 2012).

(10) $$\eta =P_{m}*Y_{m}$$

### Method to statistically validate the results

During the experiments, independent variables (parameter settings) were systematically varied, and the effect on the dependent variables (purity, yield) was reported. As boxplots are particularly suitable for the presentation of multivariate analyses (Backhaus et al., 2021), the results of this study are primarily presented using boxplots in combination with the distribution of the underlying data shown by data points. In this study, boxplots are displayed with boxes extending from the lower to the upper quartile and whiskers extending up to 1.5 times the interquartile range.

To evaluate the significance of different parameter settings and their impact on purity and yield in 2D and 3D products, statistical evaluation methods were employed. Since only for the test material with 22.5 wt% 2D material content all parameter settings were varied, these experiments were statistically evaluated. As the parameter ‘paddle type’ has two settings (closed and 45 mm paddles), a hypothesis test was conducted. For all other parameters, more than two parameter settings were tested; therefore, a multifactor analysis was used. For all tests, a level of significance of α = 0.05 was chosen. The evaluated data were aggregated every 10 seconds.

The basic requirements for many statistical tests are the normal distribution and homogeneity of variance of the tested data (Backhaus et al., 2021; Janczyk and Pfister, 2020). The normal distribution was tested using the Shapiro–Wilk test (Shapiro and Wilk, 1965). The data for each parameter setting of the test material with 22.5 wt% 2D material were checked for normal distribution using SciPy (scipy.stats.shapiro; Virtanen et al., 2020). The results are presented in Supplemental Materials 10 and 11, showing small *p*-values (*p* < 0.01), which indicates that the data is not normally distributed. As an additional exploratory step, the Levene test was used to test the homogeneity of variance (Bartlett, 1937) using SciPy (scipy.stats.levene; Virtanen et al., 2020). The results are given in Supplemental Material 12. Since the process parameters represent bounded data and non-normality was indicated, non-parametric tests were chosen for evaluation. Since the analysis aims to identify clearly identifiable, robust main factors, each factor was assessed in relation to the underlying other factors. Due to the repeated measurement design, the Wilcoxon signed-rank test was performed for the paddle type (Sprent, 2001; Wilcoxon, 1945) and the Friedman test for the other parameters (Friedman, 1937; Sprent, 2001). For both tests, the scipy library in Python was used (scipy.stat.wilcoxon and scipy.stats.friedmanchisquare; Virtanen et al., 2020). In addition, a conservative alpha correction (Bonferroni) was performed (Fahrmeir et al., 2016). The results are given in Supplemental Materials 13 and 14. However, it should be considered that, as the analyses focused on identifying robust main effects, no interactions between parameter settings were assessed, and each parameter was evaluated across the underlying settings of the other parameters. For further exploration, after the Wilcoxon signed-rank test, the effect strength *r* (Fritz et al., 2012) and after the Friedman test, Kendall’s *W* was determined as a measure of effect strength (Kendall and Smith, 1939; Tomczak and Tomczak, 2014). The results are given in Supplemental Material 15.

## Results and discussion

The following section discusses the main results. To ensure an equal number of trials, the evaluation of parameter settings focused on test material with 22.5 wt% 2D material content (sections ‘Impact of 2D material properties (RQ 2.1)’, ‘Impact of paddle angle (RQ 2.2)’, ‘Impact of the paddle type (RQ 2.3)’, ‘Impact of the rotational speed (RQ 2.4)’ and ‘Possibilities to identify a process optimum (RQ 3)’). The results of the statistical evaluation of varied parameter settings with 22.5 wt% 2D material content are provided in Supplemental Material 13 (paddle type) and Supplemental Material 14 (paddle angle, rotational speed and 2D material properties). Results on paddle type and rotational speed with 82.5 wt% 2D material content are compared to test material with 22.5 wt% 2D material content in section ‘Impact of 2D material content (RQ 2.5)’.

### Grammages per material and comparison of area- and mass-based purity and yield (RQ 1)

**Figure 2.**
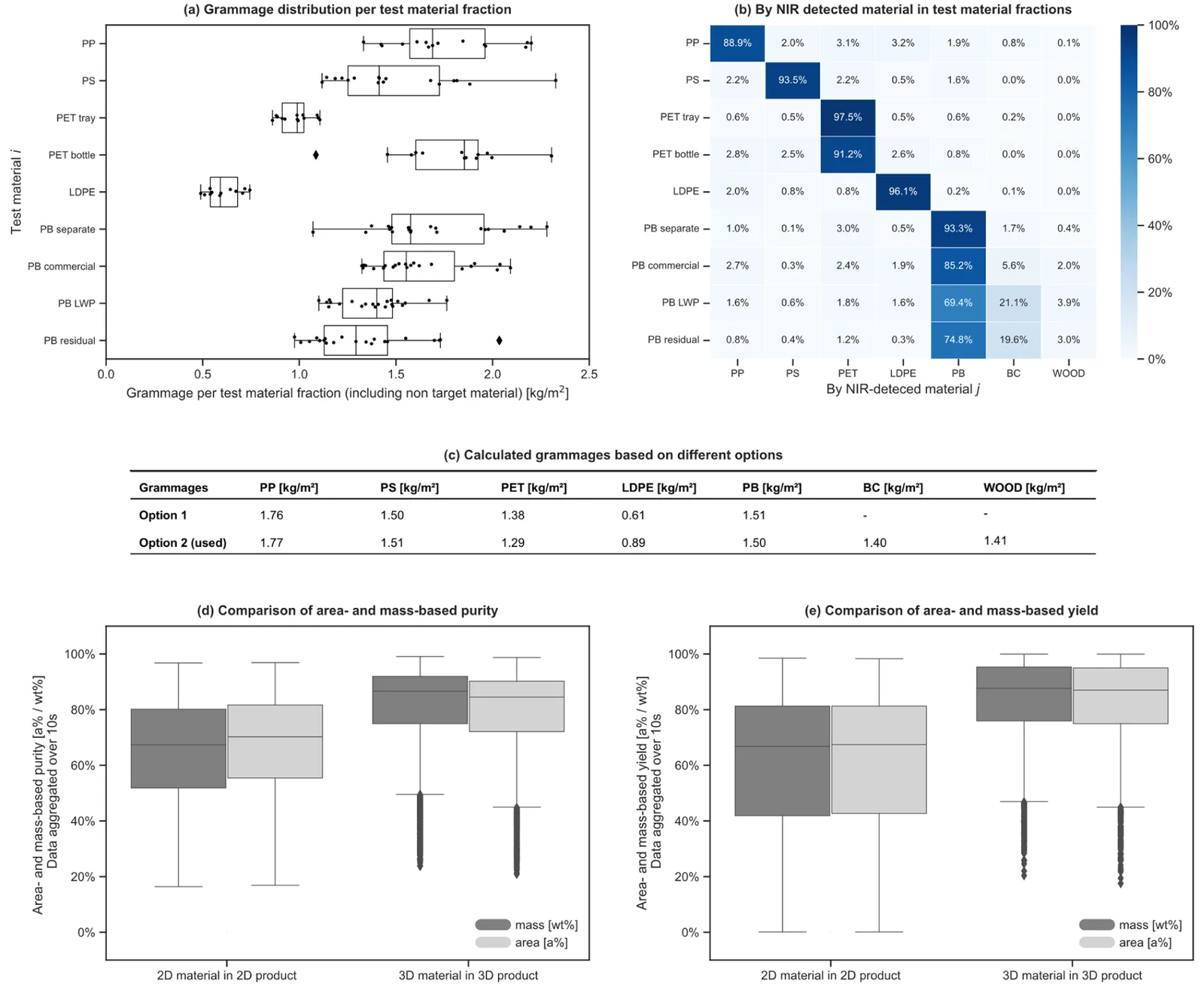
Grammages to convert area to mass flows: (a) Grammages detected for each test material by subsamples, (b) materials by NIR detected *j* in each test material fraction *i*, (c) resulting grammages calculated by equation (2) (option 1) and equation (3) (option 2), (d) comparison of the area- and mass-based purity distribution of all conducted trials (e) comparison of the area- and mass-based yield distribution of all conducted trials. NIR: near-infrared.

To obtain mass-based process indicators, grammages were determined for each test material. Figure 2(a) shows the determined grammages for all test material subsamples. Each data point represents one subsample (cf. section ‘Test material grammages’). The more heterogeneous the material fractions are, the higher the grammage variation. For example, the PS and PP test materials consisted of various articles, including cups, bottles and other types of packaging, resulting in high grammage variation. For PET tray and LDPE, the articles within each material fraction are quite similar; therefore, the grammage variation is lower. In PB from separate paper collection and commercial waste, more boards with higher grammages and less sanitary paper with lower grammages were contained than in PB from LWP and residual waste (cf. Supplemental Material 1).

Since real waste, rather than mono-fractions, was used to ensure the highest possible representativeness for industrial-scale applications, each test material fraction also contained other materials (e.g. resulting from sleeves, labels or lids). The NIR-detected composition of each test material fraction is shown in Figure 2(b). Especially in PB from LWP and commercial waste, a high content of coated PB was contained, which was detected as BC. However, since BC was classified as a 2D material, the calculated purities and yields were not affected.

Figure 2(c) shows the resulting grammages per NIR-detected material based on the options presented in section ‘Test material grammages’. As wood and BCs were only included as impurities, no test material subsamples were recorded, and no grammage calculations were possible using option 1. With option 2, it was possible to determine the grammages for the fractions of wood and BCs. Moreover, as the same test material was used to determine the grammages and conduct the trials, option 2 provided a valid method for determining the grammages per material class. With this approach, it was ensured that each material pixel detected by NIR could also represent parts from other test material fractions to a certain extent and probability. Therefore, the grammages calculated by option 2 were chosen to determine the resulting purities and yields.

Mass-based purities and yields were calculated according to equations (4) – (7). Figure 2(d) and (e) show the distribution of area- and mass-based purities and yields of all trials. The results show slightly higher 2D area-based purity and yield distributions than mass-based ones. The results correspond to the fact that 2D materials typically have lower grammages than 3D materials, resulting in lower 2D and higher 3D mass-based purities and yields. However, in this case, the use of grammages does not lead to a major distribution change of purities and yields because mainly PB was used as a 2D material (cf. Figure 1(a)). Due to the relatively high average grammage of PB compared to the 3D material, the deviation between area- and mass-based purities and yields is relatively low (maximum deviation of 2 wt% for mean values between mass-based and area-based process indicators for all trials (cf. Figure 2(d) and (e)).

### Impact of 2D material properties (RQ 2.1)

**Figure 3.**
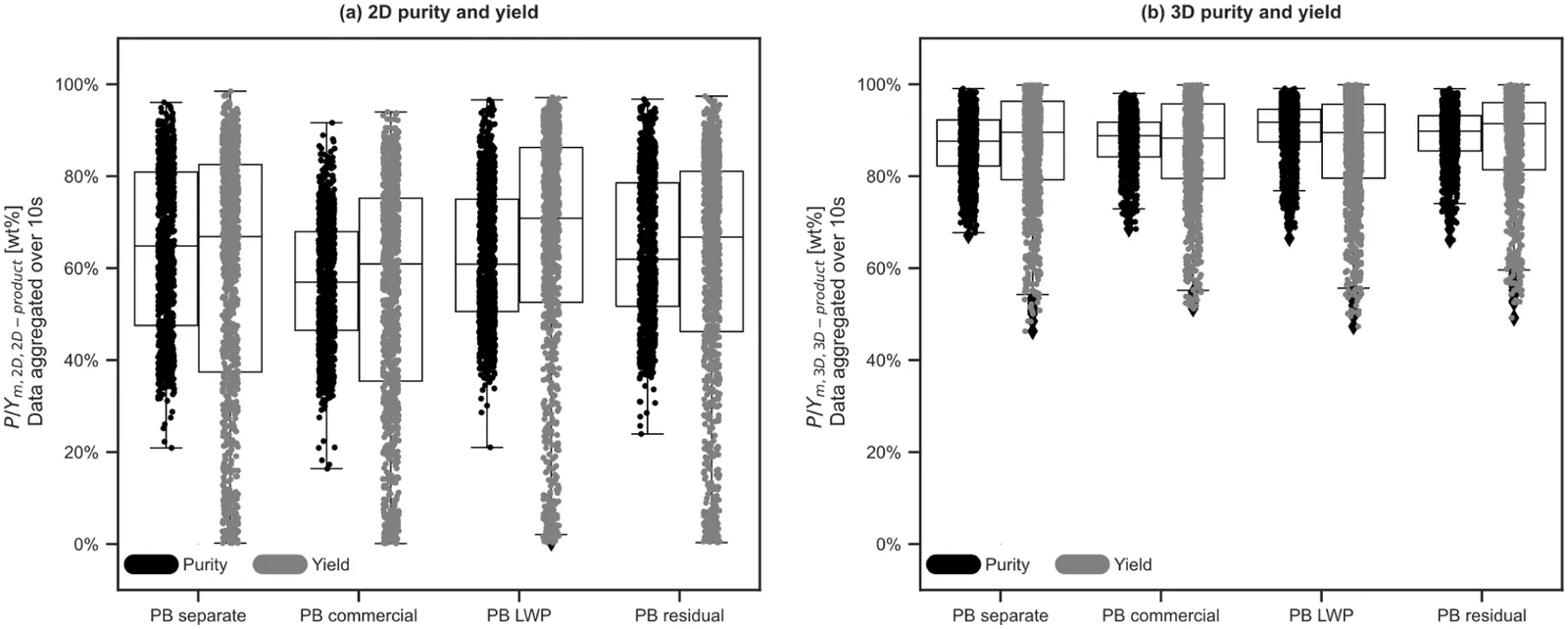
Differences of (a) 2D and (b) 3D purities and yields resulting from different 2D material properties for the test material with 22.5 wt% 2D material content (underlying parameters (for each boxplot): paddle angle, paddle type, rotational speed).

To vary the 2D material properties, PB from different origins was used. PB compositions and particle size distributions are given in Supplemental Material 1. The resulting purities and yields are shown in Figure 3(a) and (b) for test materials with 22.5 wt% 2D material content (20 wt% PB). The Friedman test showed small *p*-values (cf. Supplemental Material 14), indicating that the null hypothesis can be rejected and significant differences in the 2D and 3D purities and yields resulting from the 2D material properties can be assumed. However, Figure 3(b) shows comparably small differences resulting from different 2D material properties. PB from LWP and residual waste showed slightly higher separation efficiencies (η_2D_ and η_3D_). Possible reasons are the other material composition or particle size distribution (cf. also Supplemental Material 1). However, the high fluctuations, especially in 2D purity and yield distributions, and the relatively small differences resulting from the 2D material properties suggest that other parameters have a more significant influence on the process performance.

### Impact of paddle angle (RQ 2.2)

The paddle angle was varied between 15°, 17.5° and 20° for test materials with 22.5 wt% 2D material content (cf. Figure 1(b)). The results of the Friedman test (cf. Supplemental Material 14) indicate that the null hypothesis can be rejected and significant differences resulting from the paddle angles can be assumed. However, it should be noted that no conclusions can be drawn regarding the change in paddle angles that cause significant differences in the process indicators.

**Figure 4.**
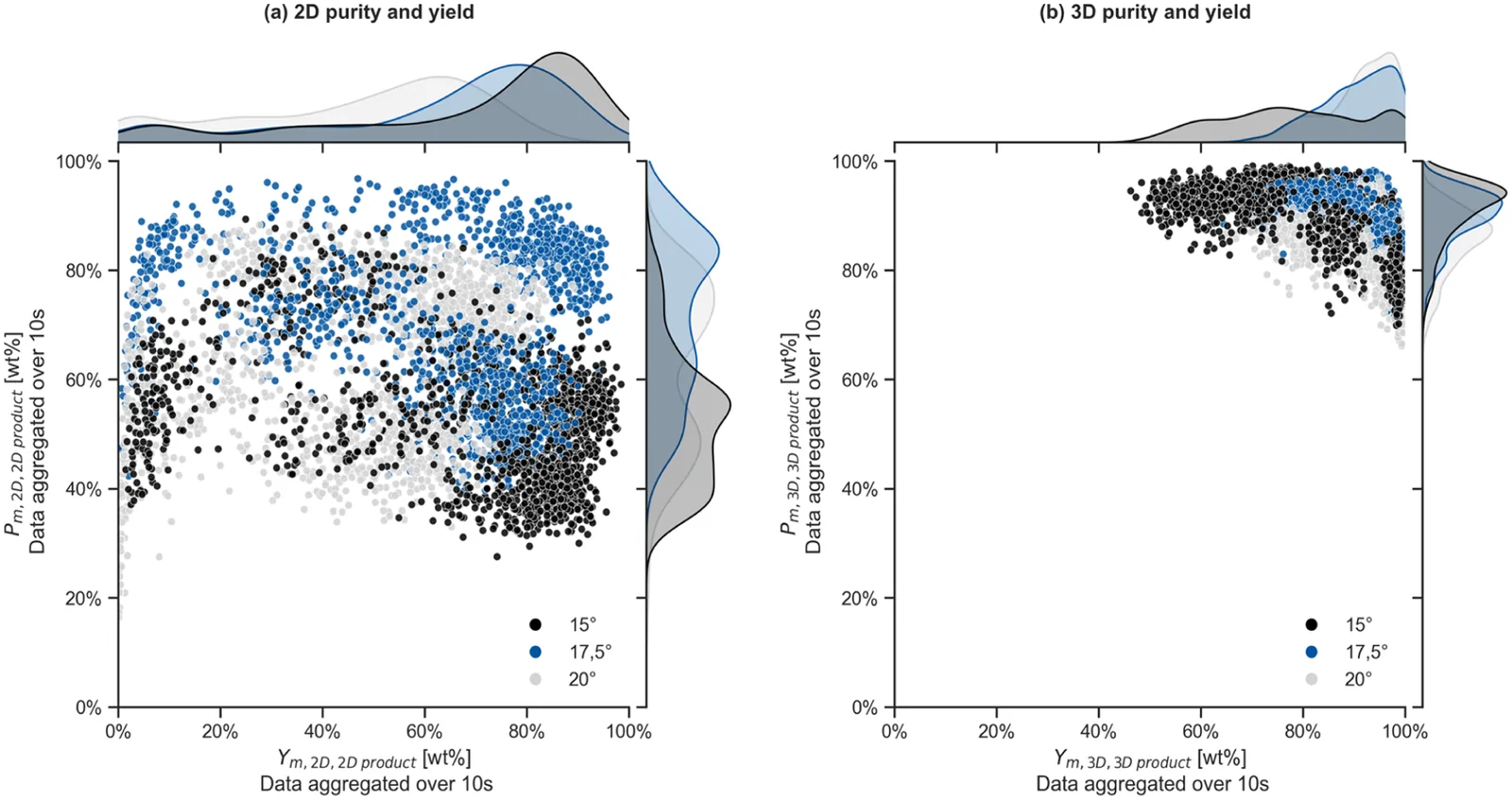
Purity and yield of (a) 2D material in 2D product and (b) 3D material in 3D product, depending on the paddle angle for test material with 22.5 wt% 2D material content (underlying parameters: 2D material properties, paddle type, rotational speed).

The resulting 2D and 3D purities and yields are shown in Figure 4. As the paddle angle increases, the 2D yield distribution decreases, whereas the 3D yield distribution increases. This is consistent with existing literature (Sigmund, 2018). For the purities, it would be expected that an increasing angle would lead to higher 2D and lower 3D purities (Sigmund, 2018). Corresponding tendencies can be observed for the 3D purity. However, the highest average 2D purity was measured at an angle of 17.5°, which was identified as a robust effect over several trials (cf. Supplemental Material 16). Looking at the material-specific yields, the yield in the 2D stream for PB continues to decrease between 17.5° and 20°. However, this trend is less pronounced for LDPE and PET (with PET trays in particular likely being ejected into the 2D stream). This may explain the lower 2D purity at 20°, as PET were classified as a 3D material. One possible explanation is that above a certain paddle angle, material properties (e.g. surface texture) exert a stronger influence on separation behaviour than the particle shape. However, since a comparably low 2D yield was measured at an angle of 20° (cf. Figure 4(a)), and the input contained only 22.5 wt% 2D material (cf. Figure 1(a)), a small material flow was directed to the 2D product. Consequently, general fluctuations and NIR misclassifications (e.g. background pollution) may have had a more pronounced influence on 2D purity, potentially leading to an underestimation of the value at 20°. In conclusion, further investigations into the impact of paddle angle on 2D purity, particularly under varying material compositions, are warranted. In general, high fluctuation ranges (for the 2D yields, up to 100%) are observed, indicating a lower influence of the paddle angle compared to other parameters, such as the tested rotational speeds.

### Impact of the paddle type (RQ 2.3)

**Figure 5.**
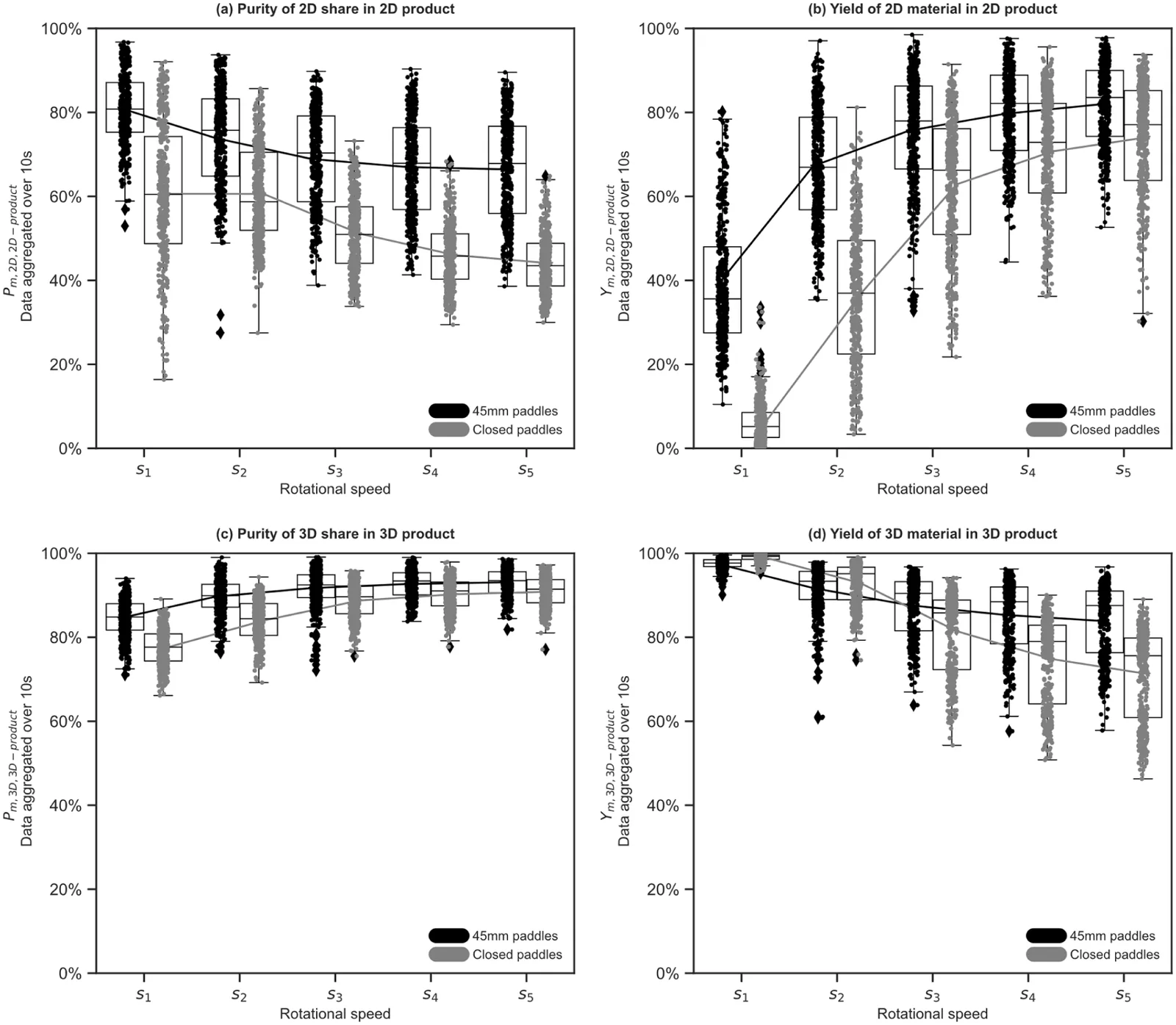
Purity and yield for 2D and 3D products, depending on rotational speed and paddle type for test material with 22.5 wt% 2D material (underlying parameters (for each boxplot): 2D material properties, paddle angle) with (a) purity of 2D share in 2D product, (b) yield of 2D material in 2D product, (c) purity of 3D share in 3D product, and (d) yield of 3D material in 3D product.

For test material with 22.5 wt% 2D material content, five rotational speeds and two paddle types were varied. Figure 5(a) and (b) show the resulting 2D and Figure 5(c) and (d) show the resulting 3D purities and yields. Except for 3D yields at low rotational speeds, the closed paddles show, on average, lower yields and purities than the 45 mm paddles. The Wilcoxon signed-rank test confirms the significantly lower process performance of the closed paddles (cf. Supplemental Material 13). However, during the trials with the closed paddles, on average 12 wt% more mass per experiment was detected than during the trials with the 45 mm paddles. The following two main reasons were identified: Firstly, due to material losses during the trials, test material fractions were weighed and refilled at regular intervals. However, water losses also occurred. Since the closed paddles were tested after the 45 mm paddles, a part of the increased mass can be attributed to the material refills. Secondly, all fines <45 mm were only detected during the trials with the closed paddles and could lead to a higher mass. However, the 2D material content, expressed as a percentage of the total area per experiment, remains relatively constant, with a standard deviation of 3.7 wt% across all 22.5 wt% 2D material trials. Process indicators were also tested to be robust against minor deviations. Additionally, lower average 2D and 3D yields and purities were observed for the closed paddles. In conclusion, although higher mass flows were detected, the generated data indicate that the closed paddles show lower overall process performance than the 45 mm paddles in material streams with a certain fine material content.

The lower process performance of the closed paddles could be explained by the higher content of fines, which would be consistent with existing literature (Möllnitz et al., 2021). Another possible reason is that closed paddles cause the material (especially the 2D material) to be ‘adsorbed’ onto the paddles (material is attracted to the paddles by the rotational movement of the paddles) and therefore worsen the overall process performance. With 45 mm paddles, the material is ‘whirled up’ more efficiently (the paddle movement and the sieve openings lead to better material turbulence), and particularly the 2D discharge is improved. Videos of the conducted trials within the ballistic separator confirm the assumption that 45 mm paddles lead to higher stress of the material than closed paddles.

### Impact of the rotational speed (RQ 2.4)

Since five rotational speeds were compared, the Friedman test was conducted, which showed significant differences for 2D and 3D purities and yields (cf. Supplemental Material 14). On average, increasing the rotational speed leads to lower 2D purities and higher 2D yields (cf. Figure 5). 3D purities increase with increasing rotational speed, whereas the 3D yields decrease. If the mean values of purities and yields are linked, asymptotic curves are observed. For the 2D material, the separation efficiency increases by 24 wt% between *s*_1_ and *s*_3_ and by only 2 wt% between *s*_3_ and *s*_5_. For the 3D material, the overall separation efficiency decreased by 8 wt% (Δη_3D,s1–s3_ = −4 wt% and Δη_3D,s3–s5_ = −4 wt%). The results indicate that the entire material on the paddles must be subjected to a sufficiently high rotational speed to achieve an effective separation effect between 2D and 3D materials. After a sufficiently high rotational speed is applied (*s*_3_), further increases do not lead to measurable differences in separation efficiency.

### Impact of 2D material content (RQ 2.5)

**Figure 6.**
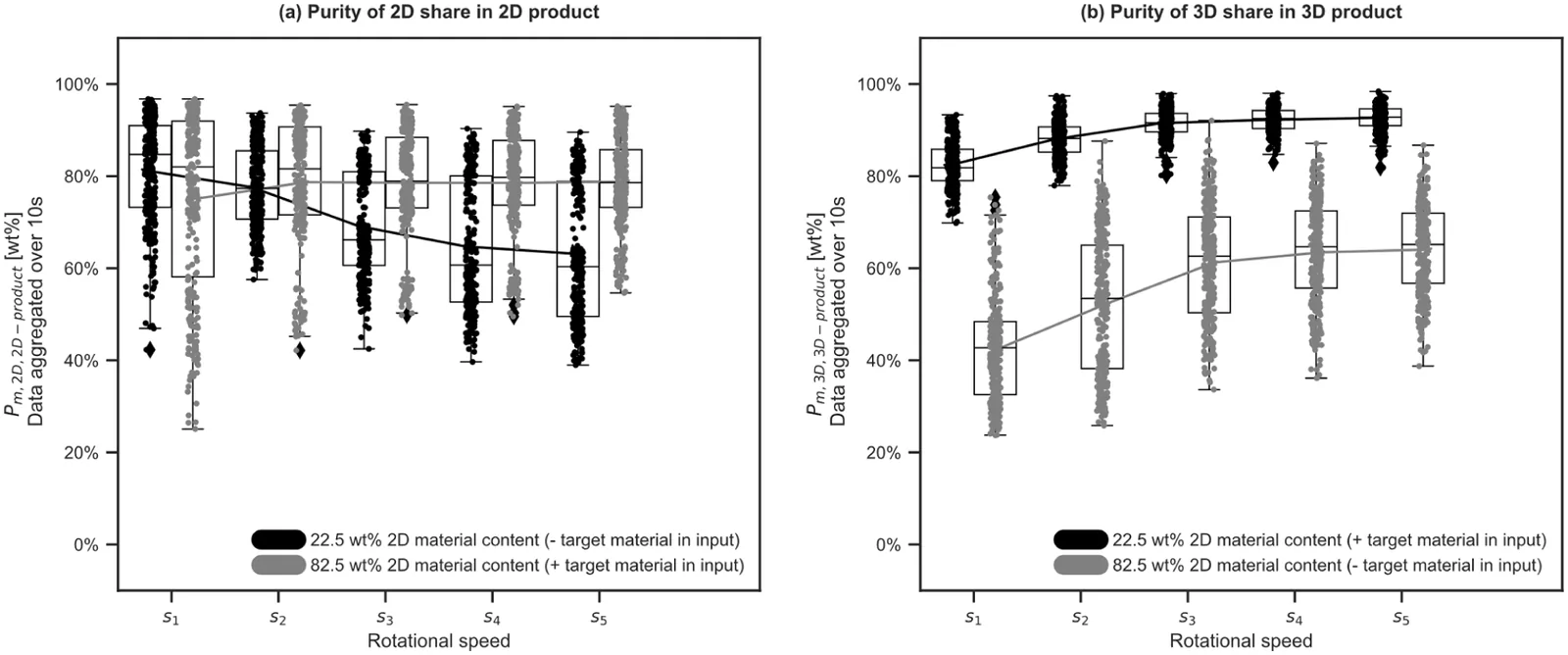
Purity of (a) 2D share in the 2D product and (b) 3D share in the 3D product for test material with 22.5 wt% and 82.5 wt% 2D material content in the input at a paddle angle of 17.5° (‘+/− target material in input’ indicates for better orientation if the respective 2D material content leads to higher or lower target material shares in the input for 2D and 3D purity, respectively; underlying parameter (for each boxplot): 2D material properties, paddle type).

In Figure 6, distributions of purities are given for the test material with 22.5 wt% and 82.5 wt% 2D material content at a paddle angle of 17.5°. The results show that higher target material contents in the input (2D/3D) lead, on average, to higher purities in the respective product stream. These results are consistent with studies that have investigated the relationship between target material content in the input and purity in the product stream from SBS units (Kroell et al., 2024b; Küppers et al., 2020). For 3D purities of material with 82.5 wt% 2D material content in the input, asymptotic curves with respect to the rotational speed were observed, similar to those with 22.5 wt% 2D material in the input. This finding is consistent with the results of Lasch et al. (2025a), who reported similar trends for 3D purities with respect to paddle speed for mixed commercial waste. This suggests that certain effects are reproducible, regardless of the material type or the proportion of target material.

### Possibilities to identify a process optimum (RQ 3)

**Figure 7.**
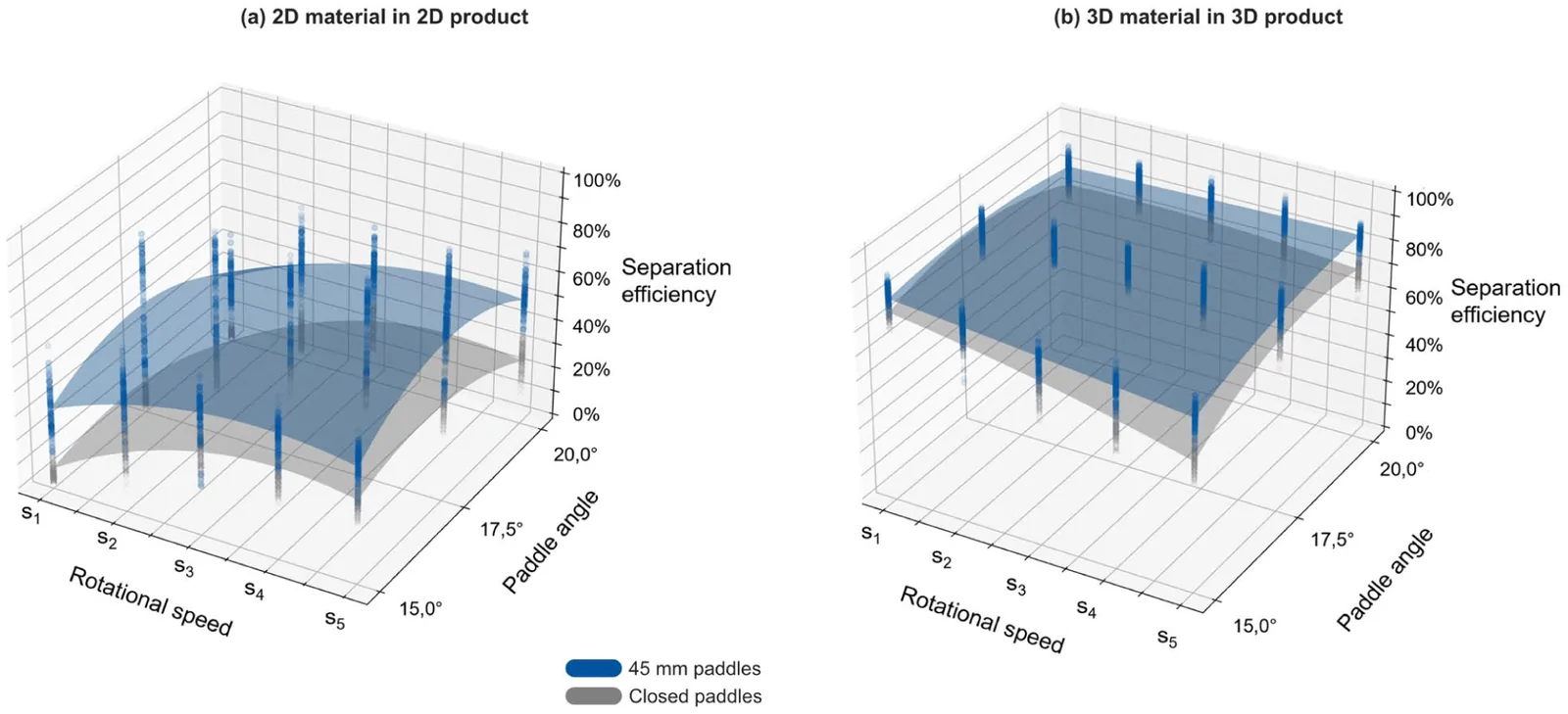
Separation efficiency for all trials with 22.5 wt% 2D material depending on rotational speed, paddle angle and paddle type (data aggregated over 10 seconds) for (a) 2D material in the 2D product and (b) 3D material in the 3D product (underlying parameter: 2D material properties).

To identify a process optimum, the separation efficiency for all trials with 22.5 wt% 2D material in the input according to equation (10) was calculated (cf. Figure 7(a) and (b)). Only results from the test material with 22.5 wt% 2D material were plotted, as for this material, all parameter settings were varied. Due to comparably small effect, no differentiation was made according to the 2D material properties (cf. section ‘Impact of 2D material properties (RQ 2.1)’). The resulting plot given in Figure 7 shows a 3D scatter plot of the aggregated separation efficiencies for each experiment. The fitted surface represents a second-order polynomial regression, modelling the relationship between the three independent variables: rotational speed, paddle angle and paddle type and the dependent variable: separation efficiency. For a better context, TCs for all trials with 22.5 wt% 2D material are given in Supplemental Material 17. However, the TCs should be interpreted with care, as it is purely based on sensor data and include the detection of multi-material components (cf. also Figure 2).

Weighing purity and yield equally, the highest point of Figure 7(a) and (b) marks the identified process optimum for 2D material in the 2D product (45 mm paddles, 17.5°, *s*_5_) and 3D material in the 3D product (45 mm paddles, 15°, *s*_1_), respectively. Depending on the sorting plant, one of the two product streams or one of the process indicators (purity and yield) may be more relevant. If ballistic separators are used in the upstream LWP sorting process before SBS cascades, the high purity of the 3D product may be a significant criterion, as particle overlaps present a considerable problem during SBS (Küppers et al., 2021). However, a higher rotational speed can lead to higher wear and, therefore, higher maintenance costs. Since a further increase of rotational speed from *s*_3_ onwards shows no major performance improvements, depending on the application, the rotational speed of *s*_3_ could present a good choice for continuous plant operation of ballistic separators. The 45 mm paddle type leads to an overall improvement in separation efficiency across all tested parameter settings. However, as 45 mm paddles lead to higher cleaning costs, a trade-off between optimal separation efficiency and cleaning costs should be calculated.

The approach to identifying a process optimum shown in Figure 7 only considers the following aspects to a limited extent: (1) During plant operation, often a minimum purity for product fractions is predefined. Therefore, it must be ensured that the predefined purities can be maintained at the identified optimum operating point. (2) Both product streams are evaluated separately. This means that a single optimum operating point cannot be determined. Therefore, an alternative approach is introduced. Firstly, all parameter settings are identified where the achieved purities are higher than the defined minimum purities (*P_m_*,_2D,2D-product,*z*_ and *P_m_*,_3D,3D-product,*z*_). Afterwards, the maximum yield *Y*_BS,*z*_, resulting from the product of the 2D and 3D yield per parameter setting *z* is calculated to maximise the yields of the 2D and 3D product. The optimal experiment z* is then equal to the experiment with the maximum *Y*_BS,*z*_. It applies:

$$\begin{array}{l} \mathrm{Maximise}\;Y_{BS,z}\\ \mathrm{Subject}\;\mathrm{to}:\;P_{m,2D,2D-product,z}=\;P_{m,2D,2D-product}>\;P_{min,2D}\\ P_{m,3D,3D-product,z}=P_{m,3D,3D-product}>P_{min,3D}\\ Y_{BS,z}=Y_{m,2D,2D-product,z}*Y_{m,3D,3D-product,z} \end{array}$$

$$z^{*}=\mathrm{argmax}\{Y_{BS,e}\;|\;P_{m,2D,2D-product,z},P_{m,3D,3D-product,z}\}$$

For example, when specifying a minimum 2D purity of 70 wt% and a minimum 3D purity of 90 wt% across all 2D material properties with 22.5 wt% 2D material in the input, this results in a process optimum for the 45 mm paddles at 17.5° and a rotational speed of *s*_5_.

## Conclusion and outlook

To evaluate the process performance of a ballistic separator on an industrial scale, a parameter study was performed with packaging waste. Using SBPM, the process performance was monitored and then evaluated using calculated mass-based purities and yields. The findings highlight the complexity of the separation behaviour in ballistic separators. Based on the data generated (main trials with 22.5 wt% 2D material in the input, referring to all principles except principle 3), six process-related principles were derived:

1. All tested parameters (2D material properties, paddle angles, rotational speeds and paddle types) showed significant influences on the process performance.
2. The 2D material properties (different types of PB material) exert a proportionally small effect on purities and yields.
3. Higher target material contents in the input (22.5 wt% vs 82.5 wt% 2D material content) lead to higher purities in the respective product streams.
4. Higher paddle angles lead, on average, to lower 2D (Δ*Y*_2D,15°–20°_ −21%) and higher 3D yields (Δ*Y*_3D,15°–20°_ +13%).
5. Regarding the paddle type, in the material stream used, which contained a proportion of fine material, the 45 mm paddles demonstrated higher separation performance compared to the closed paddles.
6. Larger differences in process performance were observed between the rotational speeds *s*_1_ and *s*_3_ (Δη_2D,*s*1–*s*3_ +24%) than between *s*_3_ and *s*_5_ (Δη_2D,*s*3–*s*5_ +2%), leading to the conclusion that a certain rotational speed must be reached to achieve sufficient material separation. Beyond this speed, no significant further improvement in process performance was measurable.

However, several limitations should be considered when interpreting the results. Firstly, the data basis comprised area-based sensor measurements that were converted into mass-based indicators using grammage values. As a result, the transferability of absolute values requires further validation. Secondly, the investigation was limited to a single type of ballistic separator and potentially influential factors, such as fines, moisture content, throughput and the material input point, were not systematically assessed. In addition, some uncontrolled parameters may have influenced the results (such as differences in fine content between the two tested paddle types) and interactions between the tested parameters were not investigated. Therefore, the results are transferable only to a limited extent. Thirdly, the analysis covers only a limited selection of material compositions. The results should therefore be interpreted and contextualised in view of these limitations.

Together, the identified process principles lead to the identification of optimal operating points using curve fitting models to maximise 2D and 3D separation efficiencies (combination of purity and yield). An additional application-oriented optimisation strategy was introduced in which purities were predefined, and yields of both product streams were maximised. This framework is transferable to other aggregates and process chains, offering a generalisable approach for process optimisation. The identified process principles and optimisation strategies support, on the one hand, process planning, especially for parameters that cannot be dynamically adjusted during plant operation. For example, the selection of 45 mm paddles could be justified by the higher process performance compared to closed paddles, despite the increased cleaning effort. On the other hand, a process understanding of parameters that can be dynamically adjusted during plant operation, such as rotational speed, can facilitate real-time adjustments and enhanced process control. Therefore, the resulting process understanding contributes to the development of automated control strategies for ballistic separators. Ultimately, improved process understanding, optimisation and automation support more efficient packaging waste sorting plants, leading to a more resilient circular economy.

## Supplemental Material

sj-pdf-1-wmr-10.1177_0734242X261433694 – Supplemental material for Assessing the process performance of ballistic separators in packaging waste sorting plants using sensor-based process monitoring Supplemental material, sj-pdf-1-wmr-10.1177_0734242X261433694 for Assessing the process performance of ballistic separators in packaging waste sorting plants using sensor-based process monitoring by Alena Maria Spies, Tabea Scherling, Annika Ludes, Bastian Küppers, Nils Kroell, Karoline Raulf and Kathrin Greiff in Waste Management & Research
