# Supplementary material for: Assessing the process performance of ballistic separators in packaging waste sorting plants using sensor-based process monitoring
Source: Waste Manag Res. 2026 Apr 11;44(9):1603–19. doi: 10.1177/0734242X261433694 (PMC13530357; doi:10.1177/0734242X261433694)
Supplement: Supplementary file 1 [file sj-pdf-1-wmr-10.1177_0734242X261433694.pdf]

Supplementary material

Assessing the process performance of ballistic separators in packaging waste sorting plants using sensor-based process monitoring

Alena Maria Spies[1], Tabea Scherling[1], Annika Ludes[2], Bastian Küppers[2], Nils Kroell[1,2], Karoline Raulf[1], Kathrin Greiff[1]*

[1]Chair of Anthropogenic Material Cycles (ANTS), RWTH Aachen University, Wüllnerstraße 2, 52062 Aachen, Germany

[2]STADLER Anlagenbau GmbH, Max-Planck-Str. 2, 88361 Altshausen, Germany

1 Methodical approach and documentation

(a) Sorting catalog description

Fraction	Definition
**Graphic paper**	By type of use, these include printing and writing papers, including fine papers
**White PB**	White paper and board
**Brown PB**	Brown paper and board
**PB composites**	Material combinations of paper or board with one or more non-paper material components, without differentiation according to separability or proportions
**Sanitary paper**	Papers with low basis weight, serving hygienic or sanitary purposes as textile substitute, single-use (tissue)
**Impurities**	Materials that do not contain paper

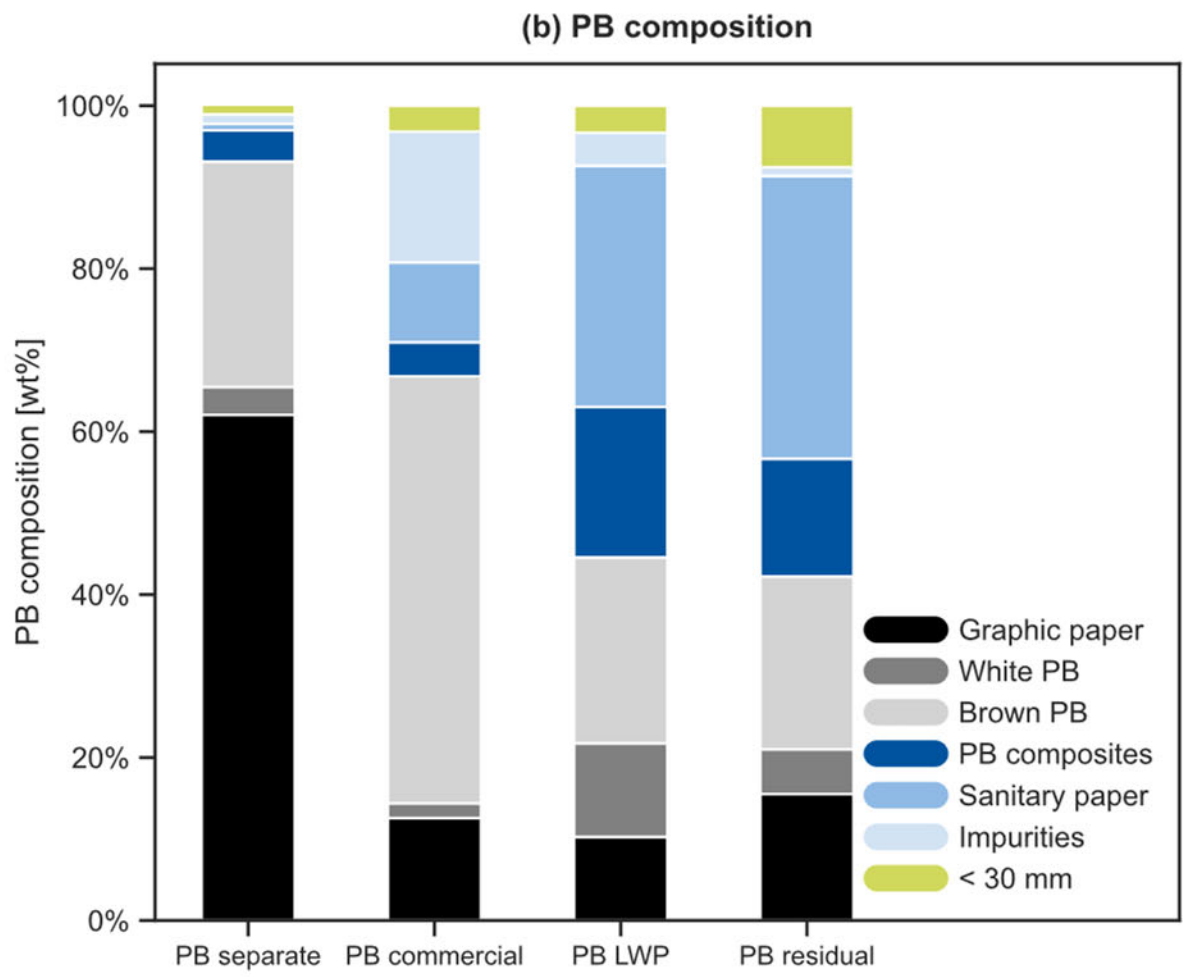

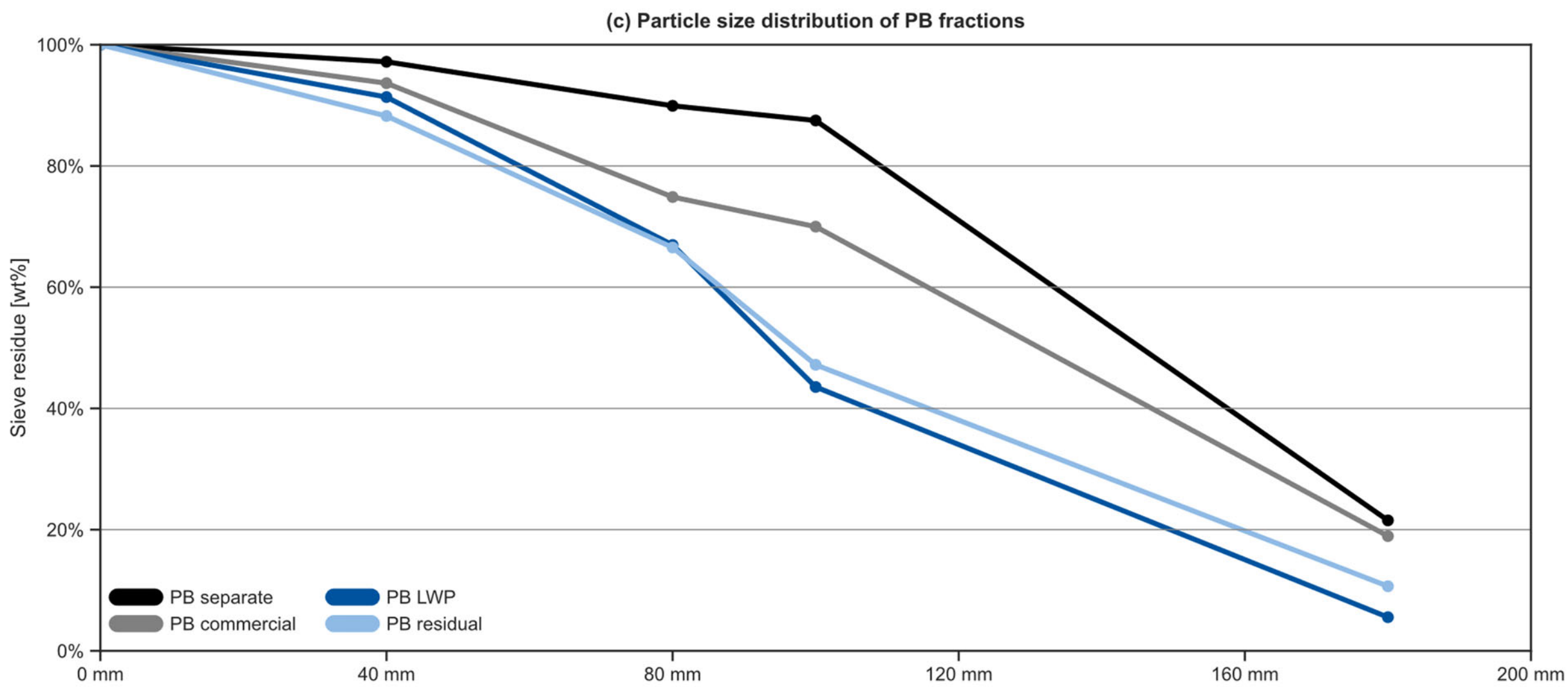

Supplementary material 1. Characterization of PB from different origins used as 2D material to vary the 2D material properties (a) sorting catalog, (b) composition based on manual analyses, (c) particle size distribution.

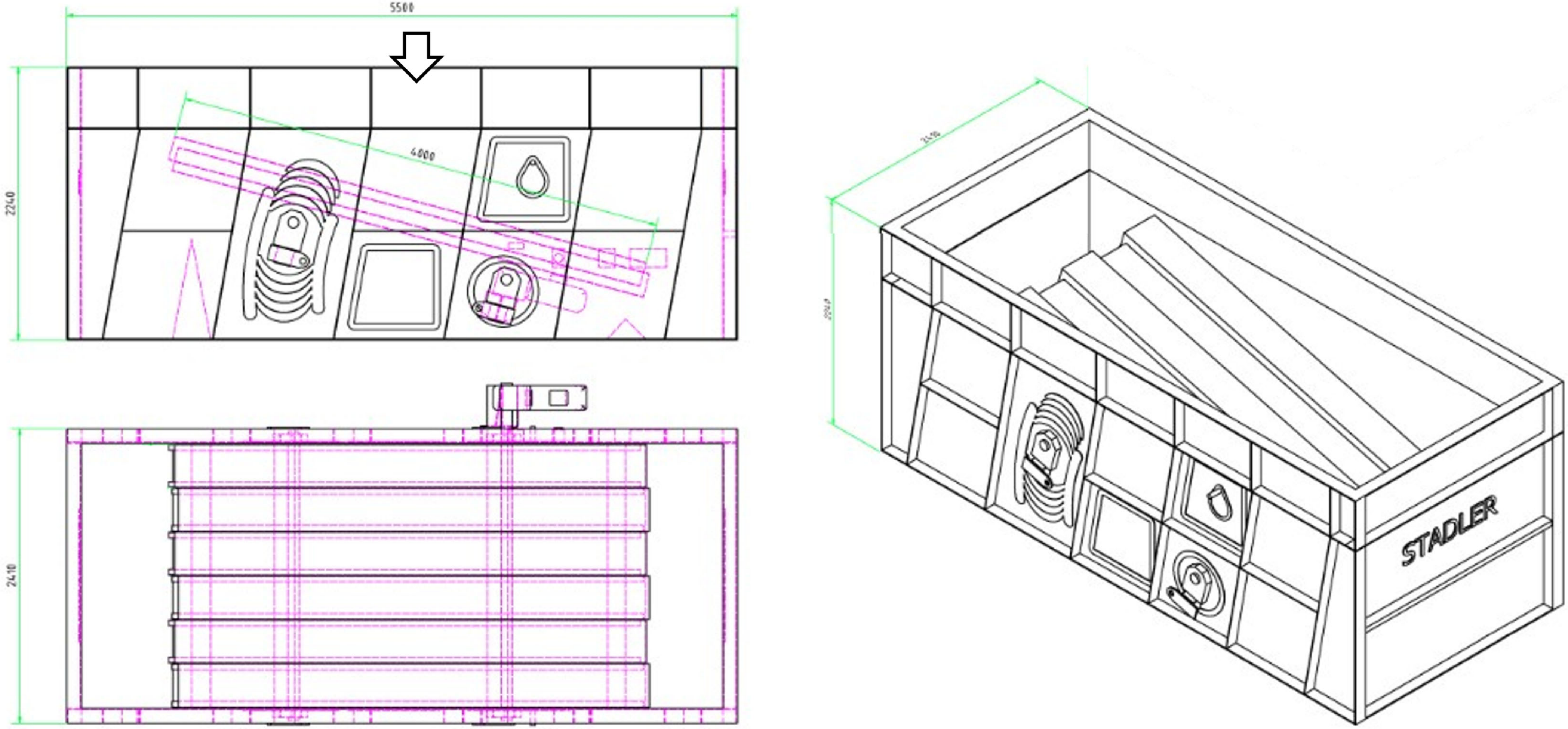

Supplementary material 2. Schematic computer-aided design (CAD) drawing of the ballistic separator STT2000_6 used for the conducted trials (all dimensions in mm).

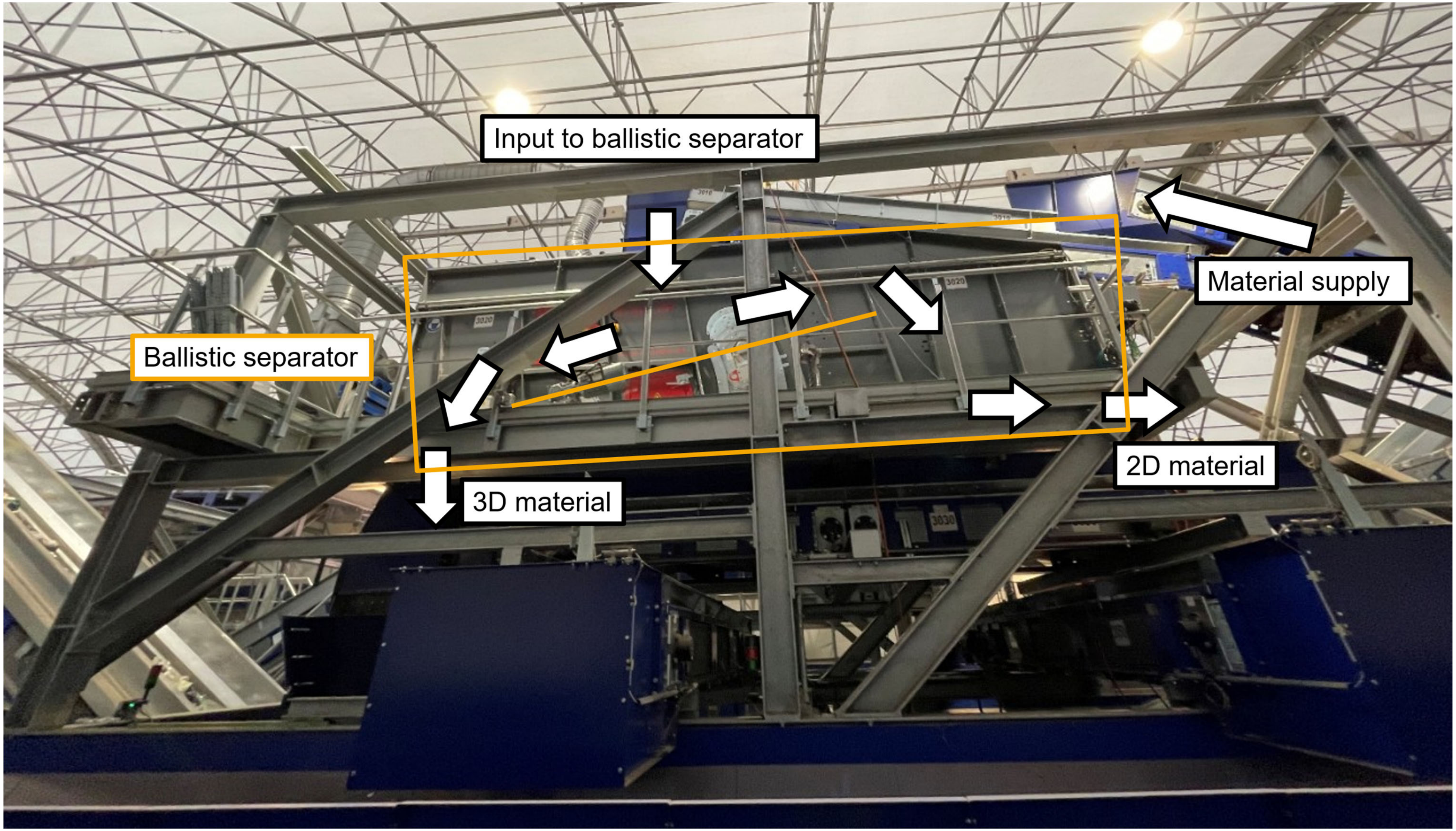

Supplementary material 3. Photograph of the ballistic separator STT2000_6 used for the conducted trials, indicating the material input and output streams.

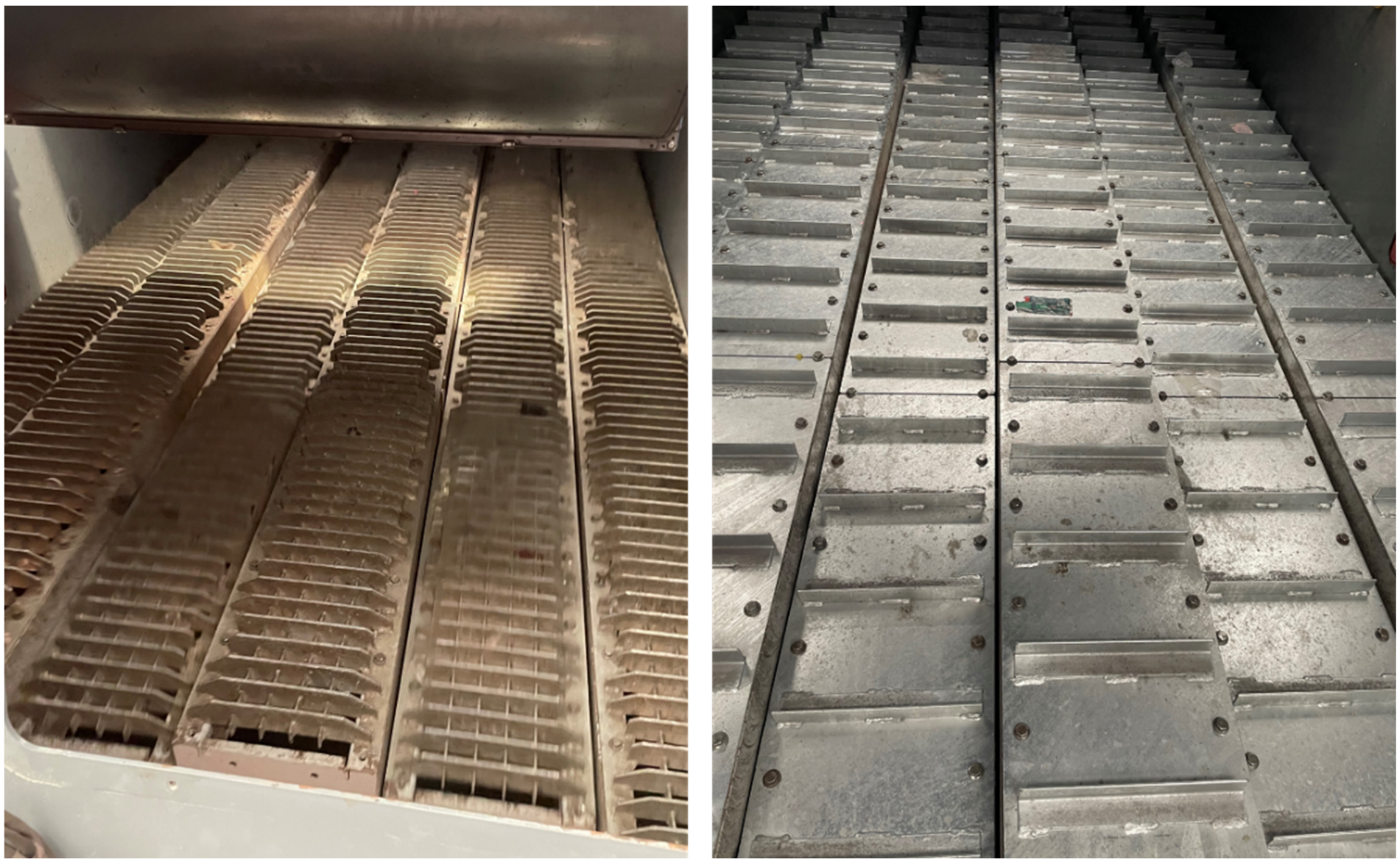

Supplementary material 4. Different paddle types. Left: 45 mm paddles (paddles with 45 mm openings), right: closed paddles

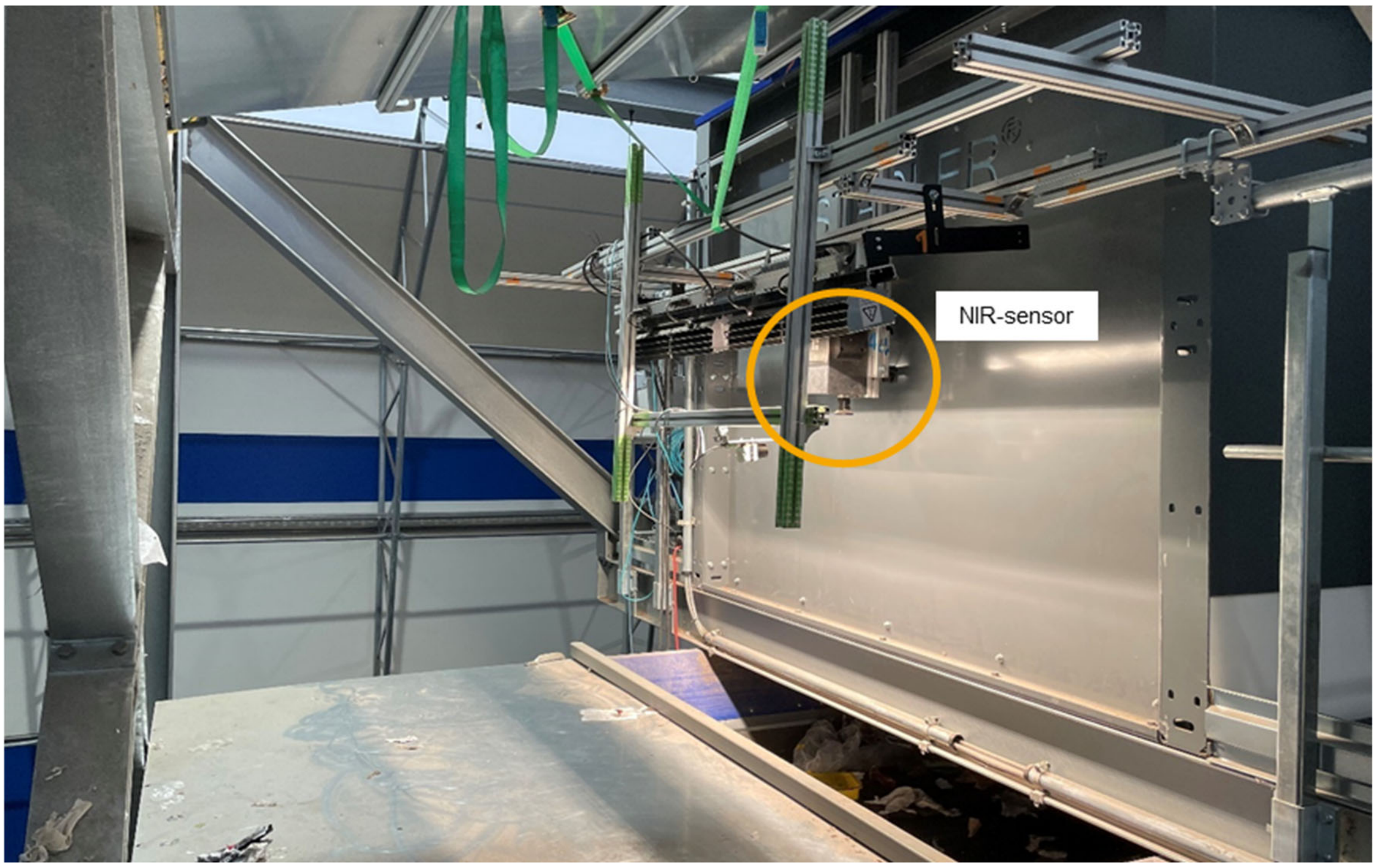

Supplementary material 5. Picture of the NIR-sensor above the 2D product stream.

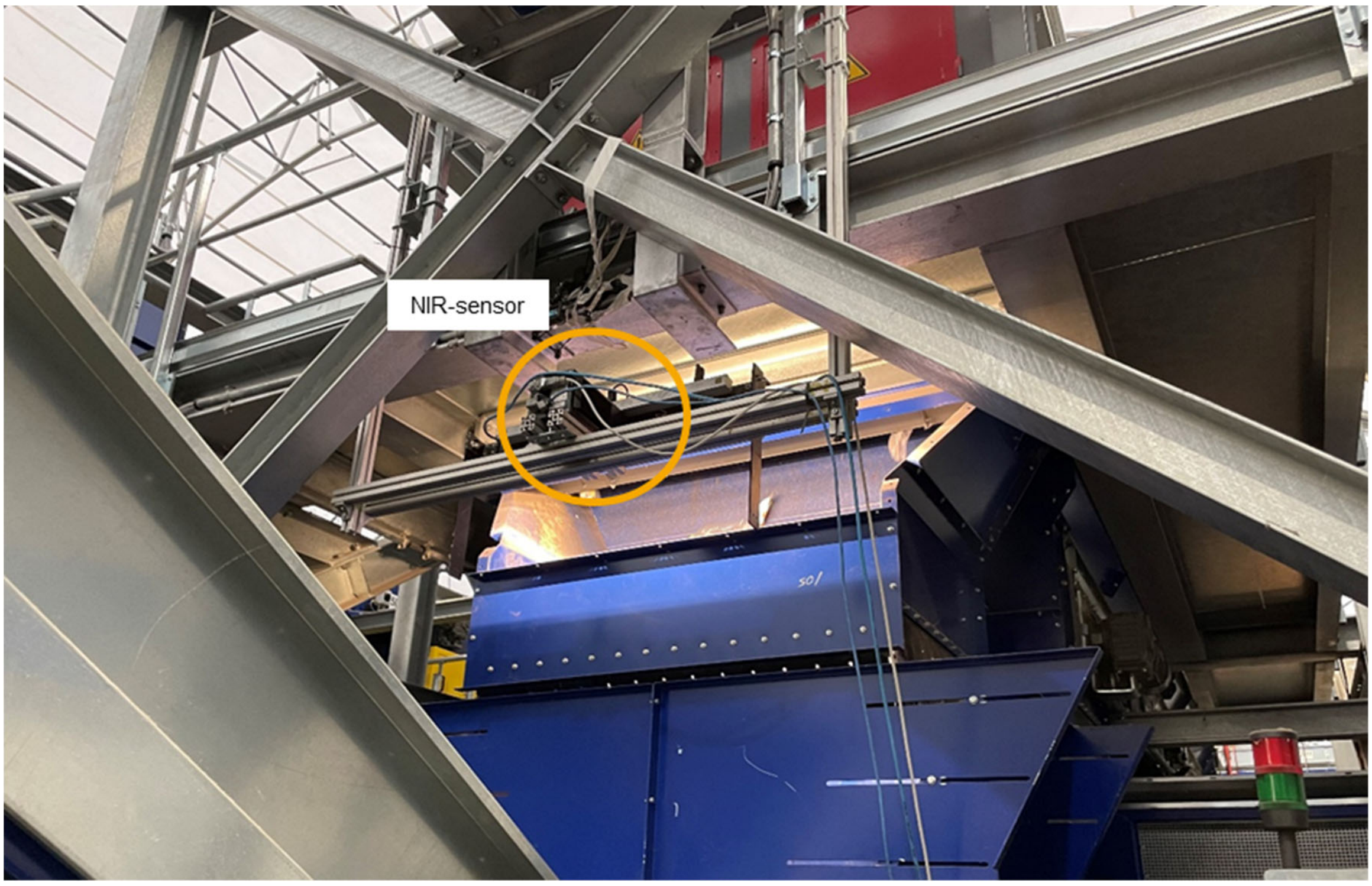

Supplementary material 6. Picture of the NIR-sensor above the 3D product stream.

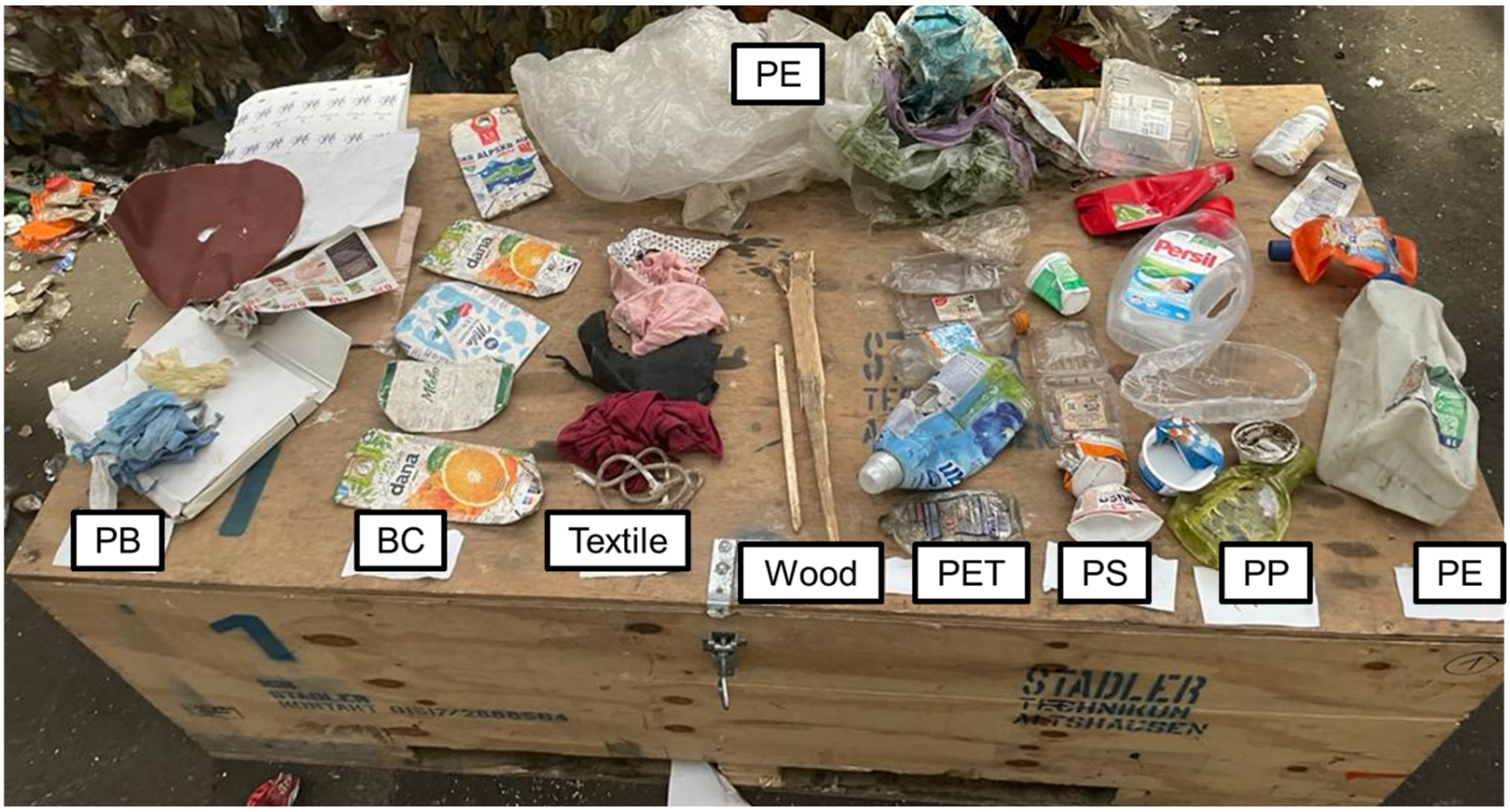

Supplementary material 7. Used material for training of NIR-sensor classification.

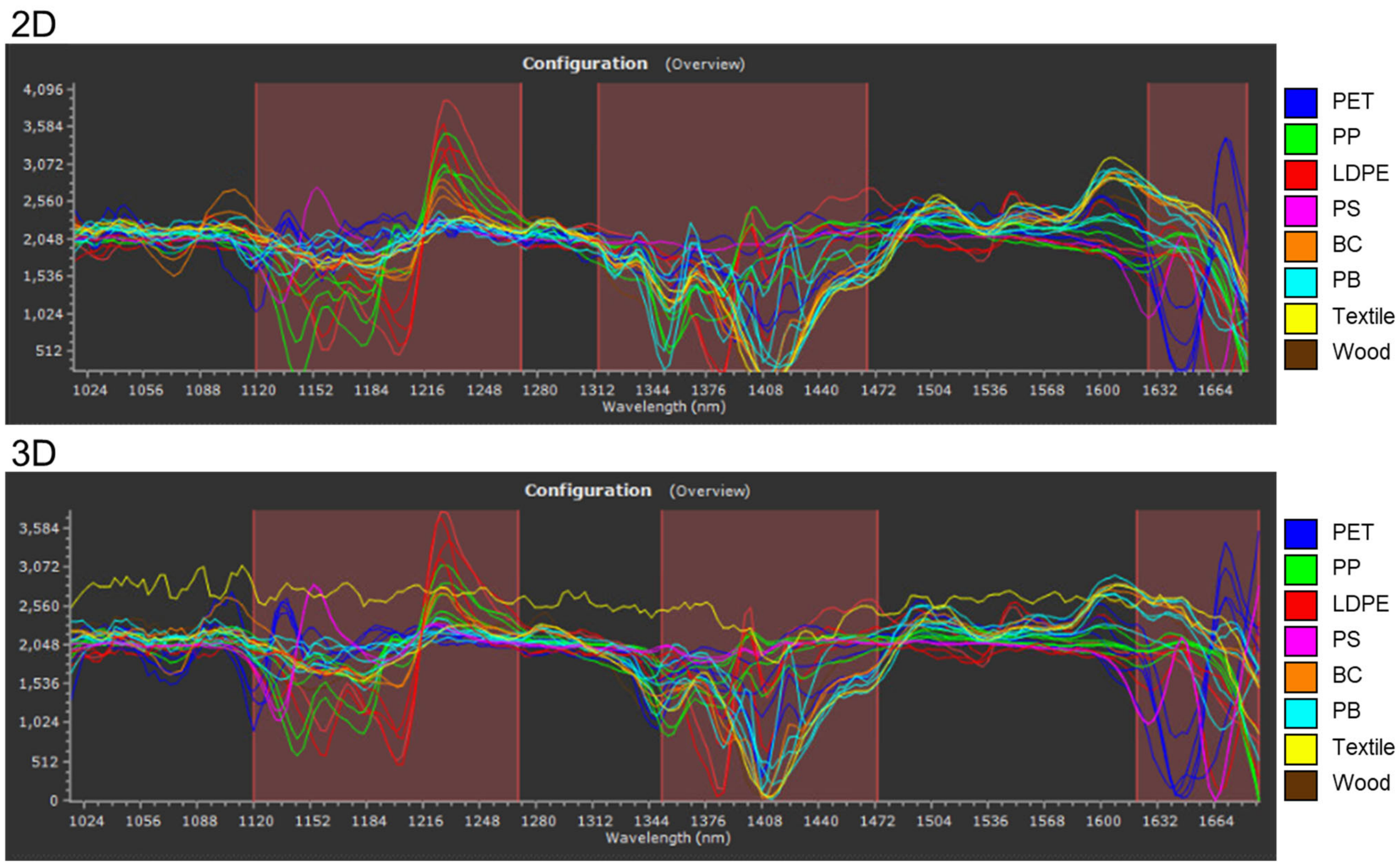

Supplementary material 8. Near-infrared spectra of the trained models.

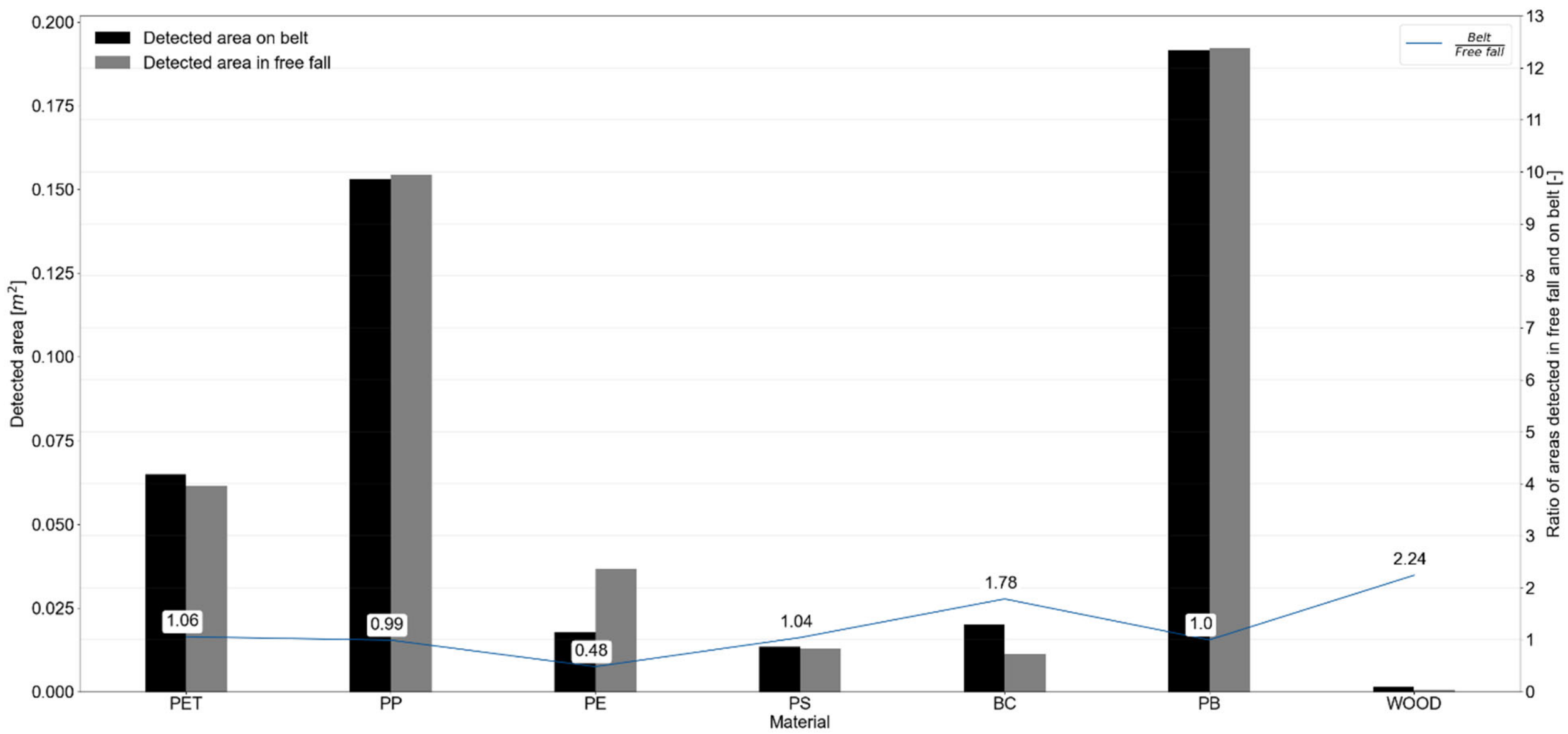

Supplementary material 9. Areas detected by NIR in free fall (3D) and NIR above a belt for single test materials objects from different material fractions.

2 Statistical evaluation

Independent variable (parameter setting)	Dependent variable	Varied parameter	Statistic Shapiro-Wilk test	p-value
Paddle angle	$P_{m,2D,2D\ product}$	17.5	0.96	$5.4\cdot10^{-20}$
Paddle angle	$P_{m,2D,2D\ product}$	20.0	0.95	$4.0\cdot10^{-21}$
Paddle angle	$P_{m,2D,2D\ product}$	15.0	0.97	$2.3\cdot10^{-17}$
Paddle angle	$Y_{m,2D,2D\ product}$	17.5	0.86	$6.3\cdot10^{-34}$
Paddle angle	$Y_{m,2D,2D\ product}$	20.0	0.93	$1.1\cdot10^{-25}$
Paddle angle	$Y_{m,2D,2D\ product}$	15.0	0.83	$6.2\cdot10^{-37}$
Rotational speed	$P_{m,2D,2D\ product}$	s_1	0.94	$6.1\cdot10^{-18}$
Rotational speed	$P_{m,2D,2D\ product}$	s_2	0.98	$1.1\cdot10^{-09}$
Rotational speed	$P_{m,2D,2D\ product}$	s_3	0.97	$2.0\cdot10^{-12}$
Rotational speed	$P_{m,2D,2D\ product}$	s_4	0.95	$1.3\cdot10^{-15}$
Rotational speed	$P_{m,2D,2D\ product}$	s_5	0.94	$3.0\cdot10^{-18}$
Rotational speed	$Y_{m,2D,2D\ product}$	s_1	0.89	$7.8\cdot10^{-24}$
Rotational speed	$Y_{m,2D,2D\ product}$	s_2	0.98	$1.1\cdot10^{-09}$
Rotational speed	$Y_{m,2D,2D\ product}$	s_3	0.96	$5.8\cdot10^{-15}$
Rotational speed	$Y_{m,2D,2D\ product}$	s_4	0.95	$2.8\cdot10^{-16}$
Rotational speed	$Y_{m,2D,2D\ product}$	s_5	0.95	$2.7\cdot10^{-17}$
Paddle type	$P_{m,2D,2D\ product}$	45 mm paddles	0.98	$7.7\cdot10^{-19}$
Paddle type	$P_{m,2D,2D\ product}$	Closed paddles	0.96	$1.4\cdot10^{-22}$
Paddle type	$Y_{m,2D,2D\ product}$	45 mm paddles	0.92	$6.3\cdot10^{-32}$
Paddle type	$Y_{m,2D,2D\ product}$	Closed paddles	0.91	$7.4\cdot10^{-34}$
2D material properties	$P_{m,2D,2D\ product}$	PB_separate	0.94	$2.4\cdot10^{-20}$
2D material properties	$P_{m,2D,2D\ product}$	PB_commercial	0.98	$1.5\cdot10^{-10}$
2D material properties	$P_{m,2D,2D\ product}$	PB_LWP	0.97	$1.2\cdot10^{-13}$
2D material properties	$P_{m,2D,2D\ product}$	PB_residual	0.96	$1.1\cdot10^{-15}$
2D material properties	$Y_{m,2D,2D\ product}$	PB_separate	0.91	$3.0\cdot10^{-25}$
2D material properties	$Y_{m,2D,2D\ product}$	PB_commercial	0.93	$1.6\cdot10^{-22}$
2D material properties	$Y_{m,2D,2D\ product}$	PB_LWP	0.88	$3.6\cdot10^{-28}$
2D material properties	$Y_{m,2D,2D\ product}$	PB_residual	0.91	$1.1\cdot10^{-24}$

Supplementary material 10. Results on Shapiro-Wilk test of normal distribution of purity and yield data from 2D material in 2D product for all tests with 22.5 wt% 2D material.

Independent variable (parameter setting)	Dependent variable	Varied parameter	Statistic Shapiro-Wilk test	p-value
Paddle angle	$P_{m,3D,3D\ product}$	17.5	0.94	$3.1 \cdot 10^{-24}$
Paddle angle	$P_{m,3D,3D\ product}$	20.0	0.96	$1.7 \cdot 10^{-19}$
Paddle angle	$P_{m,3D,3D\ product}$	15.0	0.90	$2.5 \cdot 10^{-29}$
Paddle angle	$Y_{m,3D,3D\ product}$	17.5	0.94	$1.5 \cdot 10^{-23}$
Paddle angle	$Y_{m,3D,3D\ product}$	20.0	0.92	$1.9 \cdot 10^{-27}$
Paddle angle	$Y_{m,3D,3D\ product}$	15.0	0.96	$4.7 \cdot 10^{-20}$
Rotational speed	$P_{m,3D,3D\ product}$	s_1	0.99	$3.2 \cdot 10^{-05}$
Rotational speed	$P_{m,3D,3D\ product}$	s_2	0.98	$5.2 \cdot 10^{-08}$
Rotational speed	$P_{m,3D,3D\ product}$	s_3	0.96	$5.3 \cdot 10^{-14}$
Rotational speed	$P_{m,3D,3D\ product}$	s_4	0.97	$3.2 \cdot 10^{-11}$
Rotational speed	$P_{m,3D,3D\ product}$	s_5	0.98	$9.2 \cdot 10^{-11}$
Rotational speed	$Y_{m,3D,3D\ product}$	s_1	0.90	$2.9 \cdot 10^{-23}$
Rotational speed	$Y_{m,3D,3D\ product}$	s_2	0.86	$1.1 \cdot 10^{-26}$
Rotational speed	$Y_{m,3D,3D\ product}$	s_3	0.91	$1.5 \cdot 10^{-22}$
Rotational speed	$Y_{m,3D,3D\ product}$	s_4	0.93	$2.9 \cdot 10^{-19}$
Rotational speed	$Y_{m,3D,3D\ product}$	s_5	0.94	$1.8 \cdot 10^{-17}$
Paddle type	$P_{m,3D,3D\ product}$	45 mm paddles	0.96	$6.6 \cdot 10^{-25}$
Paddle type	$P_{m,3D,3D\ product}$	Closed paddles	0.96	$6.9 \cdot 10^{-25}$
Paddle type	$Y_{m,3D,3D\ product}$	45 mm paddles	0.89	$1.2 \cdot 10^{-36}$
Paddle type	$Y_{m,3D,3D\ product}$	Closed paddles	0.92	$1.2 \cdot 10^{-32}$
2D material properties	$P_{m,3D,3D\ product}$	PB_separate	0.97	$2.3 \cdot 10^{-15}$
2D material properties	$P_{m,3D,3D\ product}$	PB_commercial	0.96	$8.0 \cdot 10^{-17}$
2D material properties	$P_{m,3D,3D\ product}$	PB_LWP	0.92	$5.4 \cdot 10^{-24}$
2D material properties	$P_{m,3D,3D\ product}$	PB_residual	0.95	$3.4 \cdot 10^{-19}$
2D material properties	$Y_{m,3D,3D\ product}$	PB_separate	0.90	$9.0 \cdot 10^{-26}$
2D material properties	$Y_{m,3D,3D\ product}$	PB_commercial	0.91	$1.2 \cdot 10^{-24}$
2D material properties	$Y_{m,3D,3D\ product}$	PB_LWP	0.90	$6.5 \cdot 10^{-26}$
2D material properties	$Y_{m,3D,3D\ product}$	PB_residual	0.87	$1.2 \cdot 10^{-28}$

Supplementary material 11. Results of Shapiro-Wilk test on normal distribution of purity and yield of 3D material in 3D product (data of all tests with 22.5 wt% 2D material).

Independent variable (parameter setting)	Dependent variable	Statistic Levene test	p-value
Paddle angle	$P_{m,2D,2D\ product}$	76.93	0.00
Paddle angle	$Y_{m,2D,2D\ product}$	1.50	0.22
Rotational speed	$P_{m,2D,2D\ product}$	7.19	0.00
Rotational speed	$Y_{m,2D,2D\ product}$	99.02	0.00
Paddle type	$P_{m,2D,2D\ product}$	1.20	0.27
Paddle type	$Y_{m,2D,2D\ product}$	357.68	0.00
2D material properties	$P_{m,2D,2D\ product}$	54.37	0.00
2D material properties	$Y_{m,2D,2D\ product}$	5.39	0.00
Paddle angle	$P_{m,3D,3D\ product}$	7.26	0.00
Paddle angle	$Y_{m,3D,3D\ product}$	525.79	0.00
Rotational speed	$P_{m,3D,3D\ product}$	57.42	0.00
Rotational speed	$Y_{m,3D,3D\ product}$	338.87	0.00
Paddle type	$P_{m,3D,3D\ product}$	146.35	0.00
Paddle type	$Y_{m,3D,3D\ product}$	468.97	0.00
2D material properties	$P_{m,3D,3D\ product}$	19.46	0.00
2D material properties	$Y_{m,3D,3D\ product}$	1.82	0.14

Supplementary material 12. Results of Levene-test on the homogeneity of variance for all parameter data sets (data of all tests with 22.5 wt% 2D material, p-value set to 0.00 for values < $1.1*10^{-3}$, rows with p-value > 0.05 are marked grey).

Dependent variable	Statistic Wilcoxon test	p-value
$P_{m,2D,2D\ product}$	1830	$8.2 \cdot 10^{-12}$
$Y_{m,2D,2D\ product}$	1826	$1.0 \cdot 10^{-11}$
$P_{m,3D,3D\ product}$	1776	$1.2 \cdot 10^{-10}$
$Y_{m,3D,3D\ product}$	1526	$3.4 \cdot 10^{-6}$

Supplementary material 13. Results of Wilcoxon signed-rang test for testing if the 45 mm paddles show significantly higher distributions for the dependent variables than the closed paddles (data of all tests with 22.5 wt% 2D material), all test show significant differences also when considering a Bonferroni correction ($\alpha = 1.3*10^{-2}$).

Independent variable (parameter setting)	Dependent variable	Statistic Friedman test	p-value
Paddle angle	$P_{m,2D,2D\ product}$	60	$1.0 \cdot 10^{-13}$
Paddle angle	$Y_{m,2D,2D\ product}$	68	$1.4 \cdot 10^{-15}$
Rotational speed	$P_{m,2D,2D\ product}$	83	$3.4 \cdot 10^{-17}$
Rotational speed	$Y_{m,2D,2D\ product}$	94	$1.5 \cdot 10^{-19}$
2D material properties	$P_{m,2D,2D\ product}$	33	$3.3 \cdot 10^{-7}$
2D material properties	$Y_{m,2D,2D\ product}$	48	$2.0 \cdot 10^{-10}$
Paddle angle	$P_{m,3D,3D\ product}$	60	$8.5 \cdot 10^{-14}$
Paddle angle	$Y_{m,3D,3D\ product}$	57	$3.5 \cdot 10^{-13}$
Rotational speed	$P_{m,3D,3D\ product}$	90	$1.3 \cdot 10^{-18}$
Rotational speed	$Y_{m,3D,3D\ product}$	96	$7.0 \cdot 10^{-20}$
2D material properties	$P_{m,3D,3D\ product}$	49	$1.2 \cdot 10^{-10}$
2D material properties	$Y_{m,3D,3D\ product}$	17	$7.3 \cdot 10^{-4}$

Supplementary material 14. Results of the Friedman test for a significant effect of all tested parameters (independent variable) on the dependent variables (data of all tests with 22.5 wt% 2D material), all tests show significant differences also when considering a Bonferroni correction ($\alpha = 4.2*10^{-3}$).

For further exploration, after the Wilcoxon signed-rank test, the effect strength r was calculated according to equation (1) with the z value of the standard normal distribution and the sample size n (Fritz et al., 2012). After the Friedman test, Kendall's W (Kendall and Smith, 1939) was determined as a measure of effect strength (Tomczak and Tomczak, 2014). It should be noted that different numbers of parameters were changed for each parameter setting, as the ranges were identified as relevant for sorting in packaging waste plants during preliminary trials, and no effect size was calculated for different parameter setting combinations.

$$r = \frac{z}{\sqrt{n}} \tag{1}$$

The effect strength supports the hypothesis that different 2D material properties lead to comparably small differences in process performance (cf. section 3.2). The effect strength of the paddle type suggests that this parameter has a particularly strong influence on 2D materials, reinforcing the hypothesis that 45 mm paddles increase material stress and thereby improve especially 2D material separation (cf. section 3.4). Rotational speed showed the highest effect strengths. However, it should be noted that effect strengths were calculated across all parameter settings (n = 5 for rotational speed, n = 3 for paddle angle, and n = 2 for paddle type), since these ranges were identified as the relevant operating window for packaging-waste sorting.

Independent variable (parameter setting)	Dependent variable	Type of effect strength	Effect Strenght
Paddle type	$P_{m,2D,2D\ product}$	r	0.87
Paddle type	$Y_{m,2D,2D\ product}$	r	0.87
Paddle angle	$P_{m,2D,2D\ product}$	Kendall’s W	0.75
Paddle angle	$Y_{m,2D,2D\ product}$	Kendall’s W	0.86
Rotational speed	$P_{m,2D,2D\ product}$	Kendall’s W	0.87
Rotational speed	$Y_{m,2D,2D\ product}$	Kendall’s W	0.98
2D material properties	$P_{m,2D,2D\ product}$	Kendall’s W	0.37
2D material properties	$Y_{m,2D,2D\ product}$	Kendall’s W	0.53
Paddle type	$P_{m,3D,3D\ product}$	r	0.82
Paddle type	$Y_{m,3D,3D\ product}$	r	0.58
Paddle angle	$P_{m,3D,3D\ product}$	Kendall’s W	0.75
Paddle angle	$Y_{m,3D,3D\ product}$	Kendall’s W	0.72
Rotational speed	$P_{m,3D,3D\ product}$	Kendall’s W	0.93
Rotational speed	$Y_{m,3D,3D\ product}$	Kendall’s W	1.00
2D material properties	$P_{m,3D,3D\ product}$	Kendall’s W	0.55
2D material properties	$Y_{m,3D,3D\ product}$	Kendall’s W	0.19

Supplementary material 15. Effect strength of all tested parameters (independent variable) on the dependent variables (data of all tests with 22.5 wt% 2D material).

3 Additional results

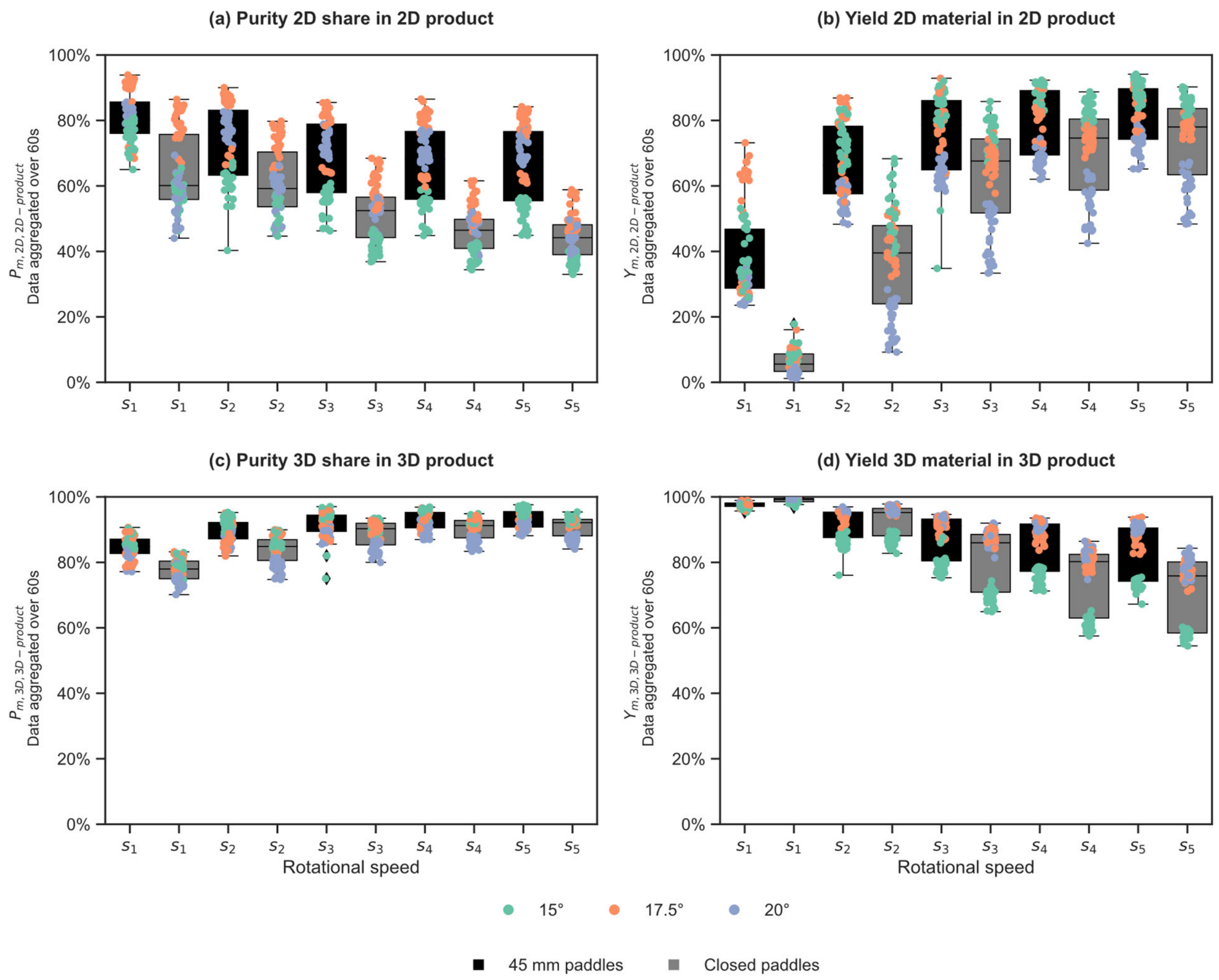

Supplementary material 16. Purity and yield for 2D and 3D products, depending on rotational speed, paddle type, and paddle angle for test material with 22.5 wt% 2D material (underlying parameter: 2D material properties).

(a) Sensor-based transfer coefficients for 2D material in 2D product

Rotational speed	Paddle angle	Transfer coefficient 2D product	
		45 mm paddles	Closed paddles
	15°	0.38	0.08
s_1	17.5°	0.47	0.07
	20°	0.29	0.03
	15°	0.74	0.51
s_2	17.5°	0.72	0.42
	20°	0.55	0.18
	15°	0.80	0.77
s_3	17.5°	0.80	0.68
	20°	0.64	0.44
	15°	0.88	0.83
s_4	17.5°	0.82	0.75
	20°	0.68	0.54
	15°	0.90	0.85
s_5	17.5°	0.84	0.78
	20°	0.72	0.59

(b) Sensor-based transfer coefficients for 3D material in 3D product

Rotational speed	Paddle angle	Transfer coefficient 3D product	
		45 mm paddles	Closed paddles
	15°	0.97	0.98
s_1	17.5°	0.98	1.00
	20°	0.98	0.99
	15°	0.86	0.87
s_2	17.5°	0.94	0.96
	20°	0.95	0.96
	15°	0.79	0.70
s_3	17.5°	0.91	0.88
	20°	0.93	0.87
	15°	0.75	0.61
s_4	17.5°	0.89	0.82
	20°	0.92	0.82
	15°	0.73	0.57
s_5	17.5°	0.89	0.77
	20°	0.90	0.79

Supplementary material 17. Transfer coefficients based on the generated sensor data and calculated masses for (a) 2D material in 2D product and (b) 3D material in the 3D product.

4 References

Fritz, C.O., Morris, P.E., Richler, J.J., 2012. Effect size estimates: current use, calculations, and interpretation. Journal of Experimental Psychology: General 141, 2–18. https://doi.org/10.1037/a0024338.

Kendall, M.G., Smith, B.B., 1939. The Problem of m Rankings. Ann. Math. Statist. 10, 275–287. https://doi.org/10.1214/aoms/1177732186.

Tomczak, M., Tomczak, E., 2014. The need to report effect size estimates revisited. An overview of some recommended measures of effect size. TRENDS in Sport Science 1, 19–25.
